# Functionally‐Graded Serrated Fangs Allow Spiders to Mechanically Cut Silk, Carbon and Kevlar Fibers

**DOI:** 10.1002/advs.202406079

**Published:** 2024-09-20

**Authors:** Gabriele Greco, Diego Misseroni, Filippo Castellucci, Nicolò G. Di Novo, Nicola M. Pugno

**Affiliations:** ^1^ Department of Animal Biosciences Swedish University of Agricultural Sciences Uppsala 750 07 Sweden; ^2^ Laboratory for Bio‐Inspired, Bionic Nano Meta Materials & Mechanics Department of Civil Environmental and Mechanical Engineering University of Trento Via Mesiano, 77 Trento 38123 Italy; ^3^ Laboratory for the Design of Reconfigurable Metamaterials & Structures Department of Civil Environmental and Mechanical Engineering University of Trento Via Mesiano, 77 Trento 38123 Italy; ^4^ Department of Biological Geological and Environmental Sciences—University of Bologna via Selmi 3 Bologna 40126 Italy; ^5^ Zoology Section Natural History Museum of Denmark—University of Copenhagen Universitetsparken 15 Copenhagen 2100 Denmark; ^6^ School of Engineering and Materials Science Queen Mary University of London Mile End Road London E1 4NS UK

**Keywords:** cutting, engineering, functional structures, serration, spider silk, stress concentration

## Abstract

Before humans and allegedly any animal group, spiders developed “functionally graded toothed blades” that cut one of the toughest biological materials: silk. Here, this work reveals the importance of micro‐structured serrations in spiders’ fangs that allow these animals to cut silk and artificial high‐performance fibers, such as carbon or Kevlar. The importance of serrations revolves around the stress concentration at the interface between the fang and the fibers, resulting in a cutting efficiency superior to that of a razor blade. This efficiency is increased by the presence of pretension in the fibers and is high also for fibers with different diameters like silk, because of the serration grading that allows a smart positioning of the fiber in the optimal cutting condition. This work proposes that when the silk fiber is grasped by the fang, it slides along the serrated edge till it gets locked in the serration with a comparable size, where the load to cut is minimal. These results provide a new perspective on cutting mechanisms and set the roots for spider fang‐inspired cutting tools.

## Graphical abstract

Spiders invent serrated blades hundreds of millions of years ago that mechanically cut silk, which is the toughest biological material, as well as artificial high‐performance fibers, such as carbon and Kevlar.

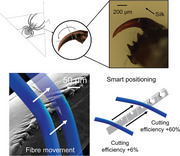

## 1 Introduction

Pushed by the challenges imposed by nature, many animals have efficiently solved biological tasks by coupling fascinating morphological traits and behaviors. Among the creatures that inspire researchers, spiders sit in a bright spot. They are capable of efficiently detecting imperceptible air flows and vibrations to locate prey or a mate,^[^ ^1^ ^]^ from which some males can efficiently flee and avoid cannibalism using a catapult action that accelerates them up to 51*g*.^[^ ^2^ ^]^ But above all, spiders are masters in spinning and weaving silks, gaining a special position in the minds of the intellectuals of every epoch.^[^ ^3^ ^]^ Spiders can produce and spin several types of silk, which present different mechanical properties.^[^ ^4^ ^]^ In particular, the strength and toughness of major ampullate silk, which outranks many natural and artificial fibers, have allowed these animals to fly to conquer many natural habitats and build robust orb webs.^[^ ^5^ ^]^ In these, spiders outsource their acoustic sensors expanding their sound‐sensitive surface area by about 10 000 times.^[^ ^6^ ^]^ Moreover, the capability of major ampullate silk to store elastic energy has allowed spiders to achieve performance otherwise impossible by using only their muscles. Recent works revealed how spiders can accelerate their body up to 80*g*.^[^ ^7^ ^]^ and lift prey 1000 times their body mass.^[^ ^8^, ^9^ ^]^ This very last work describes the interaction between the animals and the web, made of complex and disorganized networks of tough silk threads, which were promptly removed by the spider, if felt as impediments, by grasping them with the fangs and cutting.

The capacity to cut and handle silk lines is fundamental for spiders, especially for those that build webs.^[^ ^10^ ^]^ Nonetheless, the cutting mechanism has yet to receive much attention. Many authors have limited themselves in observing that the silk lines are brought into the vicinity of the mouth and broken up.^[^ ^11^ ^]^ Some authors propose that special digestive enzymes could be involved in the cutting process due to the impossibility of fangs to act like scissors.^[^ ^10^, ^12^, ^13^, ^14^ ^]^ This intuition agrees with what is commonly observed in orb weavers that ingest parts of their webs without apparent strong mechanical action of the mouth apparatus.^[^ ^10^ ^]^ The movements and the morphology of the fangs themselves are not similar to those of scissors or snipping tools.

Nevertheless, spiders possess a tool, which has been surprisingly overlooked, that may be involved in the cutting of the silk lines, and that can justify alone an exclusive mechanical action: the micro‐graded serration on the fangs. Interestingly, this particular trait of spiders has been repetitively observed in many families, but it has never been associated with a specific function,^[^ ^15^ ^]^ even though Foelix^[^ ^16^ ^]^ and Peters^[^ ^17^ ^]^ hypothesized its involvement in cutting silk lines.

Serration on fangs and teeth is not only a spider's peculiarity but is also a distinctive characteristic of other animals, such as dinosaurs,^[^ ^18^ ^]^ crocodiles,^[^ ^19^ ^]^ and sharks.^[^ ^20^ ^]^ Because of their mechanical efficiency, serrated blades, scissors, knives and swords were introduced by humans at the end of the XIX century to cut different materials (e.g., wood, steel) and food (e.g., bread, steaks). In particular, the serration in a blade is essential to efficiently cut compliant materials (such as silk), since a serrated edge can easily push its scallops into the material minimizing the required normal force.^[^ ^21^ ^]^

Thus, to be an effective tool for cutting silk, the micro‐serration on spiders’ fangs should drastically reduce the force and time required to cut fibers, thus avoiding the need for gastric enzymes to break down silk.

In this work, different experimental techniques, including custom‐made micromechanical and behavioral experiments, are combined with knowledge of the underlying mechanics and functional anatomy of spiders to understand the role of serration in the cutting process. Moreover, to better reveal and understand cutting mechanics and exclude the involvements of enzymes, we challenged the spiders to cut not only silk fibers, but also other high‐performance materials, such as carbon or Kevlar fibers. Finally, finite element (FE) simulations were performed and an analytical model was developed to prove the mechanical efficiency of graded serration in reducing the required force to cut a fiber.

Our findings lead us to propose the following cutting mechanism. The silk fiber is grasped by a fang, causing it to slide along the serrated edge of the fang until it becomes locked and then broken down in a serration of similar size.

In summary, spiders can cut silk mechanically with their serrated fangs. It is no surprise that we found such a trait in 48 araneomorph families that produce major ampullate silk and thus benefit from a tool to handle such an extreme fiber. By explaining how spiders cut, we reveal a basic engineering principle that can inspire the design of highly efficient cutting tools.

## 2 Results and Discussion

**Figure 1:**
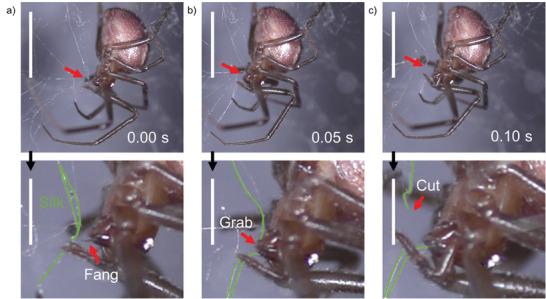
The cutting of silk by spiders. High‐speed photograph of the silk cutting sequence in a female of *Steatoda* sp. a) The spider first grabs the silk lines (here highlighted in green) with the fang to subsequently b) squeeze them between the fang and the basal part of the chelicerae to c) cut them. Scale bars of 5 mm. The panels in the lower row are enlarged about three times and the relative scale bar is 12 mm.

In previous work, we documented *Steatoda* spp. spiders hunting larger prey by lifting using pre‐tensioned silk lines.^[^ ^8^, ^9^ ^]^ When the spider is lifting the prey, the dense tangle of silk threads should impede its movements, reducing the efficiency of the process. However, this does not happen since the spider is able to cut the silk lines promptly. This cutting is demonstrated and recorded through a high‐resolution, high‐speed camera, showing how *spiders can cut silk threads in less than 0.1 s* (**Figure** **1**, Video S1, Supporting Information). The claws bring the wire close to the mouth, and the fangs open with their tips facing the thread and grab it; after which the thread seems to slide on the fang and breaks down. The observed timing and phenomenology agree with what has already been documented in the literature.^[^ ^10^, ^11^, ^12^, ^13^ ^]^ The difficulties of having this phenomenon recorded at high magnification (for example, by using a microscope) handicaps its understanding, making it hard to state if some chemical action is involved.

For these reasons, to better understand the cutting mechanism, spiders should be forced to cut different fibers in terms of materials and diameters. In this sense, Kevlar and carbon fibers are the best candidates since they are considered among the strongest and toughest artificial fibers. Moreover, these fibers are resistant to enzymes and chemical attacks, which is important to understand if a chemical action is involved in spider cutting. Thus, man‐crafted orb webs in Kevlar were used to induce spider cutting (Figure S1a,b, Supporting Information) by inserting the animal in a terrarium with these artificial webs.

During the night, spiders were recorded cutting and destroying the Kevlar threads in order to build their silk‐web (Figures S1c,d and S2a,b, Supporting Information). In particular, the animals followed the usual process to build orb webs. First, they spun the frames of the silk structures.^[^ ^10^ ^]^ Then, they removed the key structural threads in the artificial webs (Figure S2c, Supporting Information). In contrast to what happens with silk, cutting the artificial fibers proved challenging for the spiders. Unlike silk, where threads are typically cut in a fraction of a second, the artificial threads required considerable effort to cut, ≫10 s, likely involving the application of shear forces through fang movements (Video S2, Supporting Information). Eventually, the artificial fibers were cut (Video S3, Supporting Information), and the spiders constructed their web, using the leftovers of the artificial one as support (Figure S2d,e, Supporting Information).

At the same time, some other spiders were allowed to build the web in some supports where no artificial web was present. Then, some radial and spiral threads were removed and substituted with carbon fibers to stimulate spiders to also cut these artificial fibers. In a similar way to what has been described before, the animals removed the carbon fibers in the modified webs and promptly placed them at the edge of the webs. Then the animals filled the empty spaces with silk lines (Figure S3, Supporting Information).

After being cut by the spiders, the fibers’ cutting surfaces were observed with Scanning Electron Microscopy. Interestingly, the fracture surfaces of the silk and carbon fibers cut by the spiders (Figure S4a,b, Supporting Information) were similar to those broken artificially using scissors or tensile tests (Figure S5a–c, Supporting Information). Conversely, in the case of Kevlar fibers, an exhausted, and plasticized fracture surface was observed (Figures S4c and S6, Supporting Information). Plus, the fibers presented micro‐damages along their length, suggesting that the spider did not cut easily the fiber (Figure S4d, Supporting Information).

Strong mechanical actions imply powerful muscles in the chelicerae apparatus to exert the load necessary to cut such challenging fibers. Since the force exerted by a muscle is proportional to its section, we can consider the muscles of the fang (with the smaller volume) to be the limiting factor of the paw‐fang‐paw system of constraint. To investigate the biomechanics of the fang and estimate the maximum force sustainable (*F_s_*) by the muscles of the fangs in the closed position, we performed 3D µ‐tomography. The results are depicted in Figure S7 and Video S4, Supporting Information, which show that there is no separation between the fang and exoskeleton, which are connected through two flexible thickenings of the shell that determine the rotation axis. Five muscles can be identified, four flexors (white, red, violet and pink) and one extensor (blue). The tendons are anchored to the protrusions at the base of the fang.

It is very challenging to quantify the biomechanical muscle capabilities of spiders and to evaluate the forces acting on the fang apparatus,^[^ ^22^ ^]^ but a simplified calculation could still be conducted. Based on the geometrical parameters obtained from these 3D models (see Section S1, Table S13, Supporting Information), and considering the values of specific tension (force divided by the physiological cross‐sectional area) of muscles of some arachnids obtained from literature,^[^ ^23^, ^24^ ^]^ a force *F_s_* between 17 and 27 mN has been estimated, which is enough to justify a pure mechanical action in silk cutting. Such a value is comparable with the biting forces of common insects and spiders of similar size.^[^ ^25^, ^26^, ^27^ ^]^

**Figure 2:**
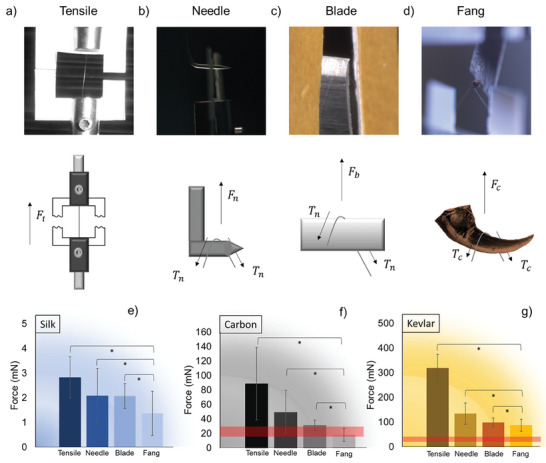
Micro‐tensile or custom‐made micro‐cutting experiments. Experiments performed to evaluate the mechanical parameters to cut the fibers. a) Tensile tests, b) 3‐points needle tests, c) 3‐points blade tests, and d) 3‐points fang tests. e) Force measured by the machine to cut silk lines with the previously mentioned setup. f) Force measured by the machine in order to cut carbon fibers with the previously mentioned setups. g) Force measured by the machine to cut Kevlar fibers with the previously mentioned setups. The red horizontal bands in subfigures f) and g) represent the range of the maximal force exerted by the spider fang computed by means of computer tomography. In the silk panel, this maximal force (17–27 mN) has not been inserted because the forces in play are much lower than it. Stars indicate that the difference is significative with *p*‐value < 0.05. The sample size for each experiment was between 9 to 22 and the analysis was performed using Excel.

However, from the behavioral experiments, we observed that i) the estimated force that a single fang can exert may not be enough to cut fibers such as Kevlar or carbon and ii) the transversal displacement applied to the silk thread is small (see Video S1, Supporting Information). Thus, spiders should own other structural features that enhance their cutting efficiency, thus reducing both the maximal force and displacement required to break the fibers. To understand this, two kinds of experiments were performed on natural (silk) and artificial (Kevlar and carbon) fibers (**Figure** **2**). The first type of experiment is a standard tensile test. These tests provided us with the mechanical properties of tested materials (Figure S7, Tables S1–S3, Supporting Information), as well as their average failure loads (Figure 2e–g, left bars). The second type of experiment was performed using a customized micromechanical experimental setup designed to mimic the spider's cutting process. Such setup resembles a sort of 3‐points test that hereafter we call a “cutting experiment” (see Experimental section). Through these experiments, we estimated the fibers breaking load (Figure 2e–g, middle and right bars), and the corresponding deflection angles (or displacement) at break. With these quantities, it was possible to calculate the stress arising within the fibers (Figures S10 and S11; Tables S4–S12, Supporting Information).

The results presented in Figure 2e–g show that the fangs are significantly more efficient than a razor blade in cutting the fibers. This difference can be ascribed to the presence of a micro‐serration on the fang since the radii of curvature of the razor blade and fang are similar. Indeed, the presence or the absence of a micro‐serration is the main difference between the fang and the razor blade, respectively (Figure S8, Supporting Information). This fact implies that spiders are favored by owning serrated fangs when cutting silk is required, in agreement with what was proposed by Peters^[^ ^17^ ^]^ and Foelix.^[^ ^16^ ^]^ Furthermore, from Figure 2e–g it is clear that the maximal force that spiders can exert, highlighted with a red band in the graphs, is enough to mechanically cut both carbon and silk fibers, but apparently not to cut Kevlar.

Contrary to what happens for crocodiles, sharks, and *Tyrannosaurus*,^[^ ^18^, ^19^, ^20^ ^]^ spider fang serration is not homogeneously spaced (Figure S12, Supporting Information). Although the mechanical response of the fiber to such serration depends on its geometry (see later), the previously presented micromechanical customized setup cannot precisely control the relative position of the fiber with respect to the serration (Figure S13, Supporting Information). This explains why the average values of cutting forces obtained with the mechanical tests are still too high to fully justify the mechanical cutting of Kevlar fibers by spiders, given the limitation on the maximum force that fang muscles can exert. However, note that multiple cuttings remain a plausible option for the spider.

**Figure 3:**
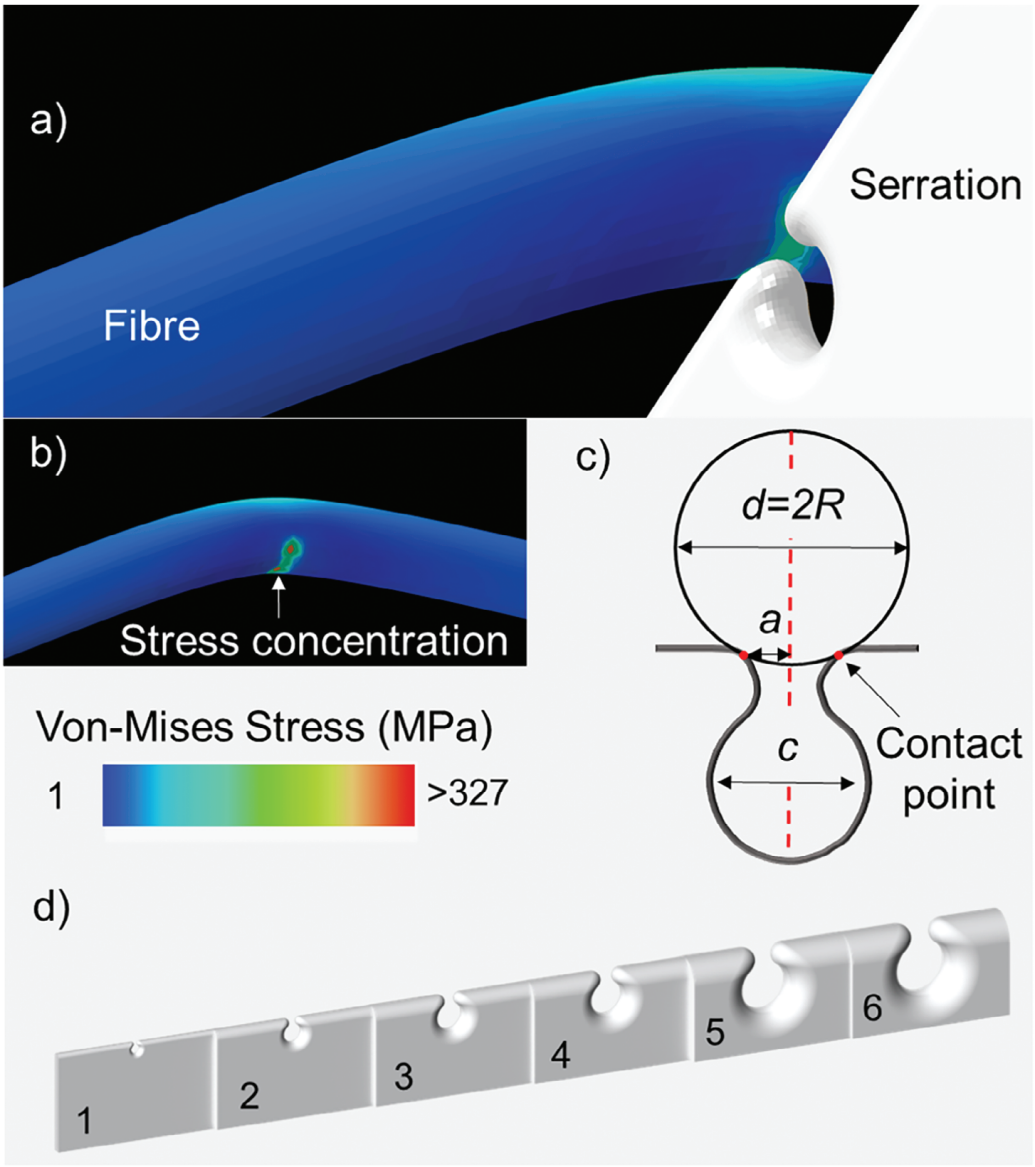
The serrations concentrate the stress at the interface between the spider fang and the fiber and improve cutting efficiency. a) Representative image of a simulation with the modelled serration used to cut the fiber. In this case *c* = 1.6. b) The same image without the serration, which depicts the stress amplification in the contact point induced by the two upper serration bulges. c) Schematic of the main geometrical parameters involved in the modelling: fiber diameter (*d*), distance between the two contact points and thus also estimation of the spacing length (*2a*), and distance between serrations (*c*) considered to be proportional to the radius of the contact region. d) 3D model of the serration with the six different considered distances *c* in the serrations that are identified by the numbers.

Systematic numerical simulations were performed to better understand the silk cutting mechanism adopted by spiders and the role played by serrations (see Experimental section for further details). **Figure** **3** highlights the pivotal role of serrations in the cutting process. When a fiber is pressed onto the fang, stress concentration is induced by the two bulges at the top of the serration (Figure 3a,b). This stress concentration initiates crack propagation, leading to the failure of the fiber. The numerical simulation results (Figure S14, Supporting Information) illustrate the impact of serrations on the cutting process. By subjecting the fiber pressed on the serrated fang to a consistent transversal displacement of 0.50 mm, the area within the fiber experiencing von‐Mises stress exceeding 326 MPa, that is, strength obtained from tensile tests (Table S1, Supporting Information), is maximized in cases *a/R* ≈ 1 (*R* is the radius of the fiber and *a* is the semidistance between the two contact points, see Figure 3c). It is noteworthy that in scenarios when *a/R* ≫ 1 no point within the fiber surpasses 326 MPa. To further investigate the role of serration in silk cutting, we have fixed the area where the von‐Mises stress is higher than 326 MPa and we measure the load necessary to achieve this value. The results (Table S14, Supporting Information) indicate that the load required to break the fiber is reduced by 80% when *a/R* = 0.96. These results strongly suggest that the optimal cutting condition is the one when the fibre and the serration have comparable dimensions.

**Figure 4:**
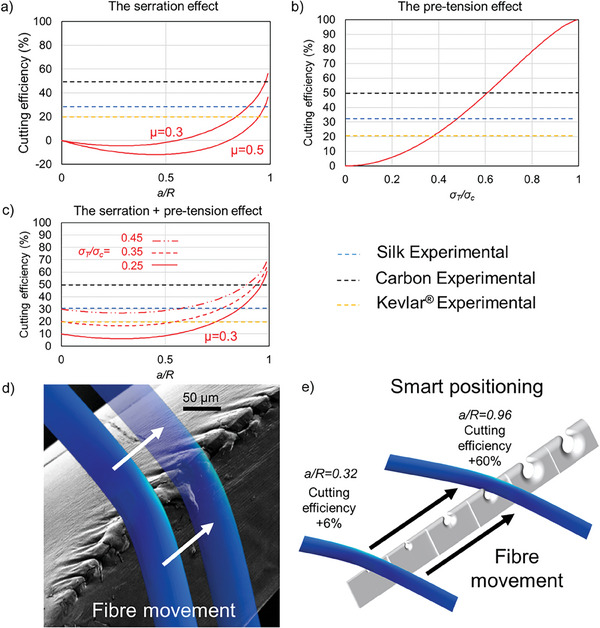
Analytical model of the cutting, smart positioning and optimal cutting. a) Serration effect: Plot of the cutting efficiency versus the *a/R* ratio at two different friction coefficients. b) Pre‐tension effect: Plot of the cutting efficiency versus relative pre‐tension stress applied by the spider for the different fiber materials. c) Serration + pre‐tension effect: Plot of the cutting efficiency versus the *a/R* ratio at different relative pre‐tension stresses, showing the effect of both the different serrations and pre‐tension stresses. Dashed colored (blue, black, and yellow) lines indicate the experimental values of the cutting efficiency for the different materials (silk, carbon fiber, and Kevlar, respectively). d) In this panel we propose a schematic of the cutting mechanism: the fiber slides along the serrated edge (SEM image of the real serration) till e) its smart positioning, interlocking in the serration where the cutting is more advantageous. Panel e) values were obtained for *µ* = 0.3 and *σ*_*T*_/*σ*_*c*_ = 0.25. The experimental data are those related to the load necessary to break the fibers obtained from Tables S6,S9, and S12, Supporting Information.

In addition to numerical simulations, cutting mechanics can also be interpreted and explained with an analytical model (see Section S2, Figure S15, Supporting Information). This considers how the serration, friction, and pretension applied by the spider on the fiber modulate cutting efficiency, here defined as

(1) $$\mathrm{Cutting}\;\mathrm{efficiency}=1-\frac{P_{ST}}{P_{0}}=1-{\left(1-\frac{\sigma_{T}^{2}}{\sigma_{c}^{2}}\right)}^{\frac{3}{2}}\;\left(\sqrt{1-{\left(\frac{a}{R}\right)}^{2}}+\mu \frac{a}{R}\right)$$

where *P_ST_* is the load to cut the fiber with serration (*P_S_* if only with the serration) and a pre‐tension (*P_T_* if only with the pre‐tension) and *P*_0_ is the critical load necessary to cut the fiber in the absence of serration and pre‐tension, here defined as control condition of negligible cutting efficiency. [Correction added on 27 November 2024 after online publication: the second term of equation 1 has been updated.] The critical stress σ_*c*_ and the pre‐tension stress σ_*T*_ are defined in Section S2, Supporting Information. If the cutting efficiency is positive the cutting is aided, by either the serration or the pre‐tension. The effect of serration is ruled by the ratio *a/R* and by the friction coefficient μ between the fang and the fibre. If cutting efficiency is negative, it means that the load required to cut the fibre is higher than *P_0_*, meaning that the condition is disadvantageous for cutting. The results predicted from the theoretical model are depicted in **Figure** **4** (see Section S2, Supporting Information for more details on the construction of the model) and have been obtained using the experimental data reported in this work. From Figure 4a is clear that the condition necessary to have an optimal cutting due to serration is *a/R* close to 1. In particular, for *µ* = 0.3, 0.5 the load to break the fiber in the presence of serration is reduced by a factor of 56%, and 36%, respectively. In general, serration has a positive effect on cutting when *a/R* > 0.54 for *µ* = 0.3 or *a/R* > 0.8 for *µ* = 0.5, suggesting that the lower the friction the sooner and the higher the positive effect of serration. Additional aid in cutting silk lines may be provided by additional tension in the fibers induced by the spiders by pulling with the legs the threads,^[^ ^28^ ^]^ as it is commonly found in cutting‐leaf ants that prior to the cutting stiffens the leaves by means of vibrations.^[^ ^29^ ^]^ Figure 4b shows the effect of pre‐tension on cutting efficiency, and it is clear that having a pre‐tension on the fiber always positively affects cutting efficiency. In particular, when $\frac{\sigma_{T}}{\sigma_{c}}=\frac{1}{2}$ the cutting efficiency is about 40%. A combined effect of pre‐tension and serration is displayed in Figure 4c, from which with a ratio *a/R* = 0.84 we obtain a cutting efficiency of 30% in the absence of pre‐tension, which can raise up to 50% by applying a pre‐tension of $\frac{\sigma_{T}}{\sigma_{c}}$ = 0.45. Overall, the analytical model aligns well with the numerical simulations’ results, that is, the optimal cutting condition is achieved when the fiber and the serration have comparable size.

The cutting phenomenon cannot be visualized in focus using light microscopy, which underscores the importance of the proposed model (SS2) and the numerical simulations in providing a potential explanation. We propose that the cutting is achieved by smart positioning the fiber to be cut along the serrated edge of the fang. Thanks to the graded serration of the spider fang and its curvature, the optimal cutting condition could be achieved just by the fiber sliding on the fang (Figure 4d,e). Thus, during cutting, the fang grasps the fiber that slides on the different serrated edges till it gets locked in the one with comparable size and thus where the cutting load is nearly minimal. This means that the presence of a functionally graded spacing between subsequent serrations (contrary to other animals^[^ ^18^, ^19^, ^21^ ^]^) permits the cutting of multiple fibers with different dimensions (such as those found in the silk threads spun by spiders). Both these aspects imply that serration is an advantageous trait for spiders and should be commonly found in these animals.

A closer look at the literature data and original data indicates that serration has been observed in 48 araneomorph families and at least three mygalomorph families.^[^ ^30^ ^]^ (Figures S16 and S17, Supporting Information). This means that the serration may have played a function even in the absence of major ampullate silk (e.g., aiding the chewing and smashing of prey). Thus, the role of serration in cutting the tough major ampullate silk may have been later acquired in Araneomorphae.^[^ ^31^ ^]^

The results reported in this article highlight that the sole mechanical action produced by spiders with their serrated fangs could be enough for cutting silk, carbon and even Kevlar fibers. Enzymes and gastric fluids may play a role in cutting mechanics, as suggested by Eberhard,^[^ ^14^ ^]^ though this does not rule out the mechanical involvement of fang serrations. Spider gastric fluids, while typically unable to rapidly dissolve major ampullate silk, are unlikely to solely induce fast cutting observed (≈0.1 s).^[^ ^32^, ^33^ ^]^ Additionally, such chemical action would not significantly affect Kevlar and carbon fibers, which spiders also cut. Thus, it remains possible that chemical enzymes weaken the fibers, but it is sure that the mechanical action that cuts them, as here demonstrated.

Finally, Figure 2 clearly demonstrates that serrated blades are more effective than non‐serrated blades in cutting high‐performance fibers like Kevlar and carbon. With the ongoing advancement of high‐performance fibers that exhibit toughness and strength comparable to native silk,^[^ ^34^, ^35^, ^36^ ^]^ we believe our findings offer valuable insights and lay the foundation for the development of spider fang‐inspired cutting tools designed to efficiently cut fibers of varying diameters.

## 3 Conclusions

Our understanding of the mechanisms that occur in nature is challenged by the complexity of the systems involved and technical limitations. Among the most captivating and understudied natural phenomena, the cutting of silk lines performed by spiders keeps awake the minds of both arachnologists and engineers. This work shows that spiders are efficiently capable of mechanically cutting silk and other highly performant artificial fibers, such as carbon and Kevlar fibers. These were selected to challenge the spiders and to better reveal and explain the cutting mechanism. By combining experimental, theoretical, numerical and biological approaches, we provide evidence that the cutting of silk lines is mechanically possible due to the presence of functionally graded fang serrations that could also allow fiber smart positioning before optimal cutting. Although this does not exclude the involvement of gastric enzymes in this phenomenon, it surely gives a solid reason for the pervasive distribution of fang serration among spiders. Here, we suggest that such a micro‐structured serration has secondarily acquired a cutting function as a morphological tool to optimize cutting mechanics by reducing the forces necessary to break up silk fibers.

## 4 Experimental Section

### Spiders and Silk Extraction

The spiders under study are the common orb‐weaver *Nuctenea umbratica* (for the interaction with artificial webs) and the tangle web spider *Steatoda triangulosa* (for the interaction with the natural web). Adult specimens were collected around the campus in Trento (Italy) and used in the cutting experiments. The silk was forcibly extracted from *N. umbratica* at ≈1 cm s^−1^. *Nuctenea umbratica* was selected because it is known to build orb webs in captivity under certain environmental conditions, that is, the presence of at least three rigid stick‐like supports. Man‐crafted orb webs in Kevlar were built using polystyrene supports (Figure S1a,b, Supporting Information) to induce spiders to cut artificial fibers. The spiders were then let inside the cage and monitored with a nocturnal vision camera during the night. At the same time, some other spiders were allowed to build the web in some supports where no artificial web was present. Then, some radial and spiral threads were removed and substituted with carbon fibers to stimulate spiders to cut these artificial fibers. In the case of experiments on spiders, according to Italian regulations on animal protection and EU Directive 2010/63/EU for animal experiments, we are not required to obtain ethical approval.

### Artificial Spider Webs

The artificial orb webs were produced with the support of a styrofoam base, from which 8 pillars were placed to elevate the web from the plane. Kevlar Technora T240_440dtex (Teijin) and Carbon C T24‐5.0/270‐E100 (SGL) fibers were used to create the main frame and the spirals. Then, the artificial fibers were glued on the frame by Super Attack glue droplets.

### High‐Speed Video

A Sony PXW‐FS5 equipped with Nikon AF Zoom‐Micro‐Nikkor 70–180 mm f/4.5–5.6 D ED lens was used to record high‐speed cutting videos. These movies were recorded at a frame rate of 240 fps (24p).

### Cutting Experiments with Spiders

In a glass terrarium (30 × 30 × 40 cm^3^) the artificial orb web structures were placed and subsequentially a small refuge was created using rolled paper. This was placed in a high corner of the cage, to provide to the spider during the day. The spider was then placed in the terrarium and recorded at night with the support of a high‐resolution Sony Camera with night visual (Sony FDR‐AX700 4K).

### Scanning Electron Microscopy

We used a FE‐SEM Zeiss Supra‐40/40VP to perform SEM microscopy. The samples were coated by using a Quorum machine T150 with the Pt/Pd 80:20 program in a reduced argon atmosphere. SEM images were used to measure serration spacing *c* used to define the initial crack length *a* in Equation (1) and reported in Figure S5, Supporting Information (right). Such values were evaluated by computing the averages and the standard deviation of several measurements conducted on different specimens.

### Mechanical Tests

Two kinds of experiments were performed on natural and artificial fibers. Such experiments were performed using two loading frame machines: a nano‐tensile Agilent UTM T150 and a mu‐strain by Messphysic. The use of two different machines was dictated by (i) the expected loads to be applied to break the different fibers (i.e., higher load for Kevlar) and (ii) space constraints. For instance, the needle‐cutting experiments were impossible with the nano‐tensile machine since there was insufficient space to mount the razor blade on its upper grip. Before the execution of the experiments reported in this article, preliminary tests were performed with both machines to verify the correspondence of the collected results. In both experiments, the samples were prepared as follows. Paper frames were obtained by cutting a square window (10×10 mm^2^) and placing double‐sided tape to attach the fibers. For spider silk, no extra glue was necessary, whereas, for carbon and Kevlar fibers, we also used super glue to fix the fibers better. In all the cases, the fibers were mounted with a bit of slack to ensure minimal pre‐stress. The diameter of the fibers (used to calculate the cross‐sectional area and thus the stress) was measured before the experiments with the support of an optical microscope at five points for each fiber and then averaged. The results are reported in Tables S1–S10, Supporting Information.

### Tensile Experiments

These experiments were performed to estimate the mechanical properties of the fibers. We used the nanotensile machine to test silk and carbon fibers, while a mu‐strain (by Messphysic) to test Kevlar fibers. The imposed test speed (displacement gauge machines) was 6 mm min^−1^ in all the mechanical tests. The nominal stress and strain were calculated, respectively, by dividing the force by the initial cross‐sectional area and the imposed displacement by the initial gauge length (taking into account the slack before the initial loading). Young's modulus was obtained by linear fitting of the initial linear elastic region of the stress‐strain curve, strength as maximal stress, ultimate strain as maximal stain and toughness modulus as the area under the nominal stress and strain curve.

### Cutting Experiments

These experiments were specifically designed to mimic the cutting mechanism used by spiders. The test is a sort of 3‐points test, where the fibers are fixed at their ends and loaded transversally with the loading machine. The setup consisted of a loading frame machine (Figure 3a) whose upper grip, the one connected with the load cell, holds different cutting elements. These were a needle (0.2 mm diameter, Figure 3b), a razor blade (Surgical Scalpel blade #10, Figure 3c), and a spider fang (glued on a steel support, Figure 3d) from an adult specimen of *Nuctenea umbratica*. For the fang, in particular, we ensured that the serration was pointing upward against the fiber. The needle was selected to have a diameter comparable to the middle part of the fang. The razor blade was selected to have a cutting edge as sharp as the one of the spider fangs (curvature radius 3.5 µm, Figure S8, Supporting Information), with the sole main difference of not having a serration. These experiments were performed for the major ampullate silk of an adult *Nuctenea umbratica*, carbon fibers and Kevlar fibers. During the execution of the experiments, the machine applied a strain (test speed of 6 mm min^−1^) and recorded the applied load until the failure of the fibers. We used the nanotensile machine to perform the cutting tests with the needle and the fang on silk and carbon fibers. We used the mu‐strain to test (i) silk and carbon fibers with the razor blade and (ii) Kevlar fibers with all three different cutting elements. The cutting loads estimated using the three different cutting elements (needle, razor blade, and fang) were compared to those obtained via standard tensile test (4 types of test in total).

### Tomography of the Teeth

We undertook microtomographic imaging of the spider fangs in the TOMCAT beamline of the Swiss Light Source.^[^ ^37^ ^]^ The used energy was 21 keV, and the distance detector‐spider was 20 cm. This was euthanized in ethanol at 70% and kept in a vial to guarantee adequate contrast. The images were pre‐elaborated with ImageJ software^[^ ^38^ ^]^ using the plug‐in “WEKA trainable segmentation” to classify the grey‐scale images into different classes. The segmentation and the 3D volumes were measured with the support of 3DSlicer, with which all the volume images were produced.^[^ ^39^ ^]^

### Simulations

We performed systematic Abaqus (Static, General) simulations to investigate the role played by the functionally graded serration in the cutting mechanism adopted by spiders. The silk fibers were modelled as 3D elements with Young's modulus and diameter, respectively, *E* = 7 GPa and *d* = 3.33 µm. Six different simulations were performed, one for each of the serration spacing *c = {1.6, 3.292, 4.782, 6.012, 8.643, 9.514}* µm (Figure S12, Supporting Information). The radius of curvature of the contact region *r* and the distance between the contact points (2*a*) are assumed to be *r* = 0.25*c. To reduce computational costs, we divided the fibers into two main regions to have a finer mesh only where necessary. The two parts were joined together using a tie constraint. In the external regions, we used a coarser mesh made of C3D10 elements (10‐node quadratic tetrahedron) with a maximum size of 0.5. Conversely, the central region was discretized by a much finer mesh made of C3D10 elements (10‐node quadratic tetrahedron) with a maximum size of 0.035. The refinement in the central region is essential in correctly estimating the stress concentration arising at the contact region between the fiber and the fang. A mesh‐sensitive study was performed to estimate the optimal mesh sizes that led to mesh‐independent results.

To better compare the real experiments, we tried to replicate the actual fang using the SEM images as a template. Such 3D objects were realized parametrically in *SolidWorks* and then imported into Abaqus for running the simulations. Since the geometry of the serration fangs used in the simulation is an approximation of the real geometry, the simulation results provide just an indication of the stress concentration induced by serrations in the fibers. The final results are shown in Figure 3 and Table S14, Supporting Information. The fangs were modelled as 3D elements with Young's modulus *E* = 10 GPa^[^ ^40^, ^41^, ^42^ ^]^ and meshed with C3D4 elements (4‐node linear tetrahedron). To obtain reasonable results and to avoid convergence issues, we reduced the mesh size to 0.01 in the vicinity of the serration, namely in the area where contact with the fibers happens.

The contact fiber‐fang was modelled using a surface‐to‐surface frictional algorithm (friction coefficient 0.3). We have assigned the master and the slave roles to the fang and the fiber surfaces, respectively. In the simulations, the fibers were constrained with two hinges at the two ends, while a constant displacement was imposed on the fang to mimic the setup of the cutting experiment. By virtue of the remarkable ductile properties exhibited by silk fibers, we have opted to employ the von‐Mises stress as a criterion for assessing failure, which is a common approach used for both fragile and compliant materials.^[^ ^43^, ^44^ ^]^

### Mapping of Serration on the Spider Tree of Life

Information regarding spider taxa for which fang serration is present was acquired by direct observation of spider specimens and by screening literature data. The presence of serration was plotted on a cladogram including all major spider groupings derived from the phylogenomic work by Kallal et al.^[^ ^45^ ^]^ The explored literature was.^[^ ^16^, ^30^, ^46^, ^47^, ^48^, ^49^, ^50^, ^51^, ^52^ ^]^ A list of spider taxa for which serration is reported in the bibliography, together with novel data obtained in this work is reported in the Excel supplementary data.

The Data were obtained from.^[^ ^15^, ^16^, ^30^, ^45^, ^46^, ^47^, ^48^, ^49^, ^50^, ^51^, ^52^ ^]^

### Statistical Analysis

To analyze the data obtained from the experiments we employed one‐way ANOVA. For each type of experiment, the sample size was between 9 and 22. No outliers were excluded from the analysis. The *p*‐value was calculated using the data analysis package in Excel.

## Conflict of Interest

The authors declare no conflict of interest.

## Author Contributions

G.G. and D.M. contributed equally to this article. Conceptualization: G.G., N.M.P. Methodology: G.G., D.M., N.D.N., F.C., N.M.P. Investigation: G.G., D.M., N.D.N., F.C., N.M.P. Funding acquisition: G.G., D.M., N.M.P. Supervision: G.G., N.M.P. Writing – original draft: G.G., D.M. Writing – review & editing: G.G., D.M., N.D.N., F.C., N.M.P.

## Supporting information



Supporting Information

Supplementary Video 1

Supplementary Video 2

Supplementary Video 3

Supplementary Video 4

## Data Availability

The data that support the findings of this study are available in the supplementary material of this article.
