# Supplementary material for: Functionally‐Graded Serrated Fangs Allow Spiders to Mechanically Cut Silk, Carbon and Kevlar Fibers
Source: Adv Sci (Weinh). 2024 Sep 20;11(44):2406079. doi: 10.1002/advs.202406079 (PMC11600254; doi:10.1002/advs.202406079)
Supplement: Supplementary file 1 — Supporting Information [file ADVS-11-2406079-s001.docx]

**Supplementary material**

**Functionally-graded serrated fangs allow spiders to mechanically cut silk, carbon and Kevlar^®^ fibres**

**Authors:**

Gabriele Greco^1,2^*^$^ & Diego Misseroni^3$^, Filippo Castellucci^4, 5^, Nicolò G. Di Novo^2^, Nicola M. Pugno^2, 6^ *

**Affiliations:**

^1^ Department of Animal Biosciences, Swedish University of Agricultural Sciences, 750 07 Uppsala, Sweden

^2^ Laboratory for Bio-Inspired, Bionic, Nano, Meta, Materials & Mechanics, Department of Civil, Environmental and Mechanical Engineering, University of Trento, Via Mesiano, 77, 38123 Trento, Italy

^3^ Laboratory for the Design of Reconfigurable Metamaterials & Structures, Department of Civil, Environmental and Mechanical Engineering, University of Trento, Via Mesiano, 77, 38123 Trento, Italy

^4^ Department of Biological, Geological and Environmental Sciences—University of Bologna, via Selmi 3, 40126, Bologna, Italy

^5^ Zoology Section, Natural History Museum of Denmark—University of Copenhagen, Universitetsparken 15, 2100, Copenhagen, Denmark

^6^ School of Engineering and Materials Science, Queen Mary University of London, Mile End Road, London E1 4NS, UK

*Corresponding authors: [gabriele.greco@slu.se](mailto:gabriele.greco@slu.se); [nicola.pugno@unitn.it](mailto:nicola.pugno@unitn.it);

^$^ These authors contributed equally

**Supplementary text**


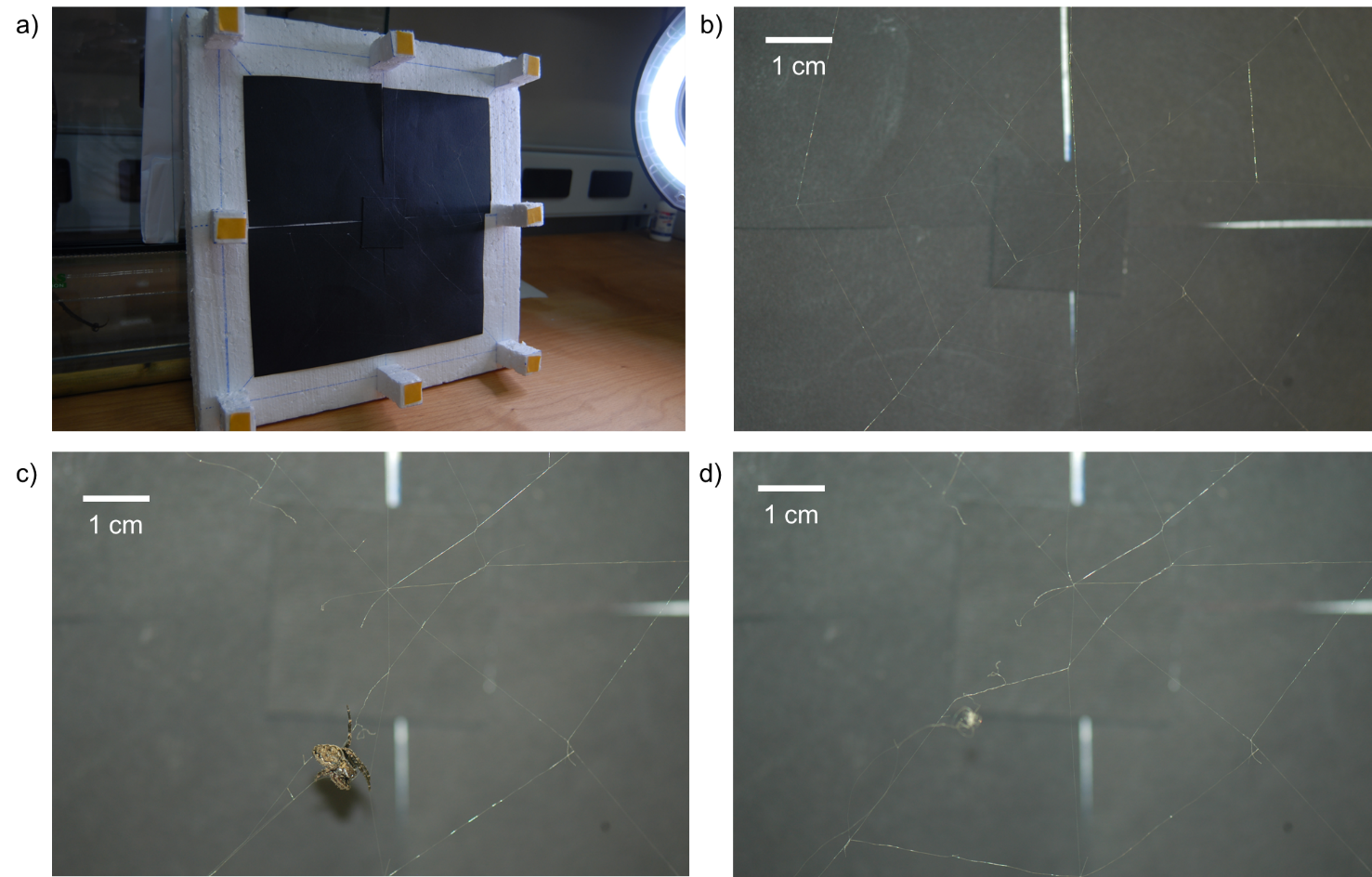


**Fig. S1. The artificial webs and the cutting of Kevlar.** a) Picture of the artificial orb web made of Kevlar^®^ used to challenge the spiders. b) Detail of the centre of such a web. c) The spider is cutting and destroying the web to build its own. d) The residual fibres of the artificial web without the spider.


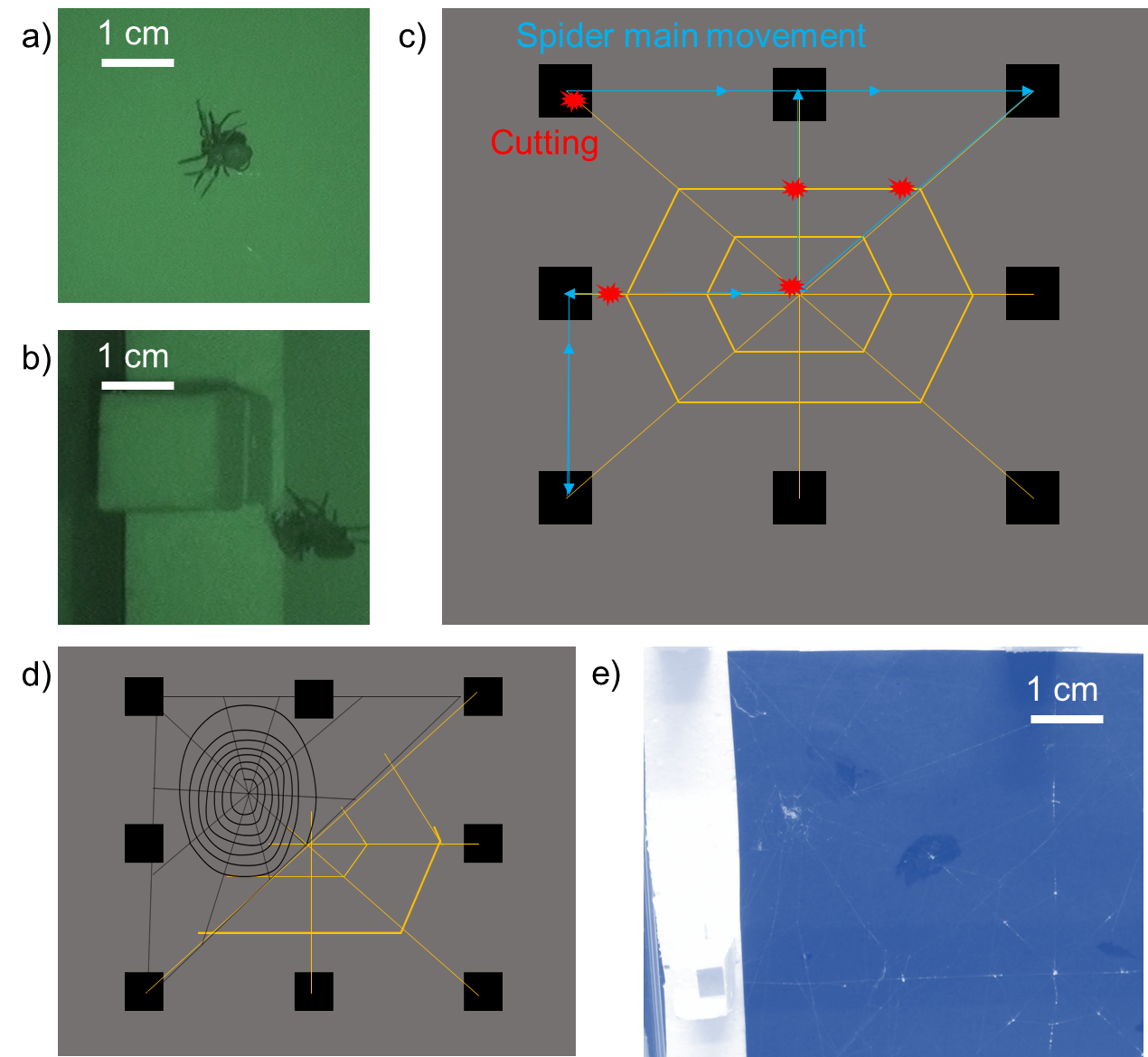


**Fig. S2. The interaction of the spiders and the artificial orb webs.** a-b) Night frames of the spider while it is cutting the artificial web threads. c) Schematic of the movement of the spider during the night, followed with a night vision camera, and the points in which the cutting was typically performed. d) After the partial destruction of the artificial web, the spiders built their own to replace it. e) Picture of the orb web built by the spider on the leftovers of the artificial one.


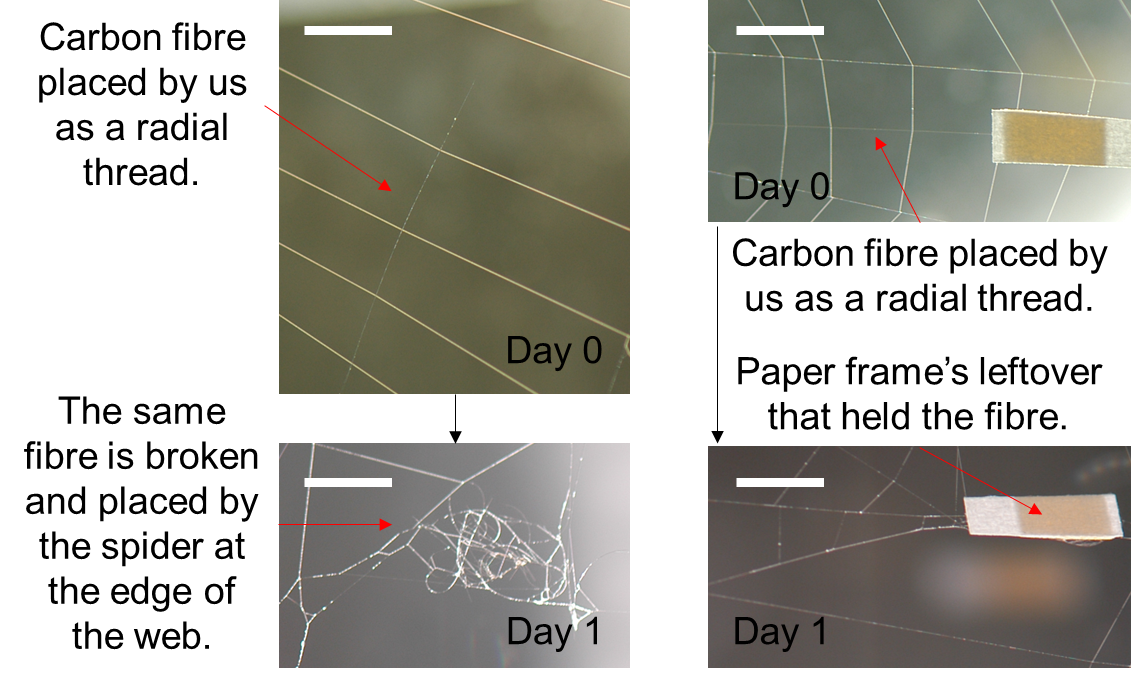


**Fig. S3. The natural spider orb webs hybridized with carbon fibres and the spider reaction.** Given the impossibility of making self-standing artificial orb webs with carbon fibres, the spiders were challenged by placing the carbon fibres (with a paper frame at the end to identify them) as radial threads. These, considered as disturbances in the webs, were broken and removed by the spiders that placed them at the edges of the webs. Scale bars are 5 m.


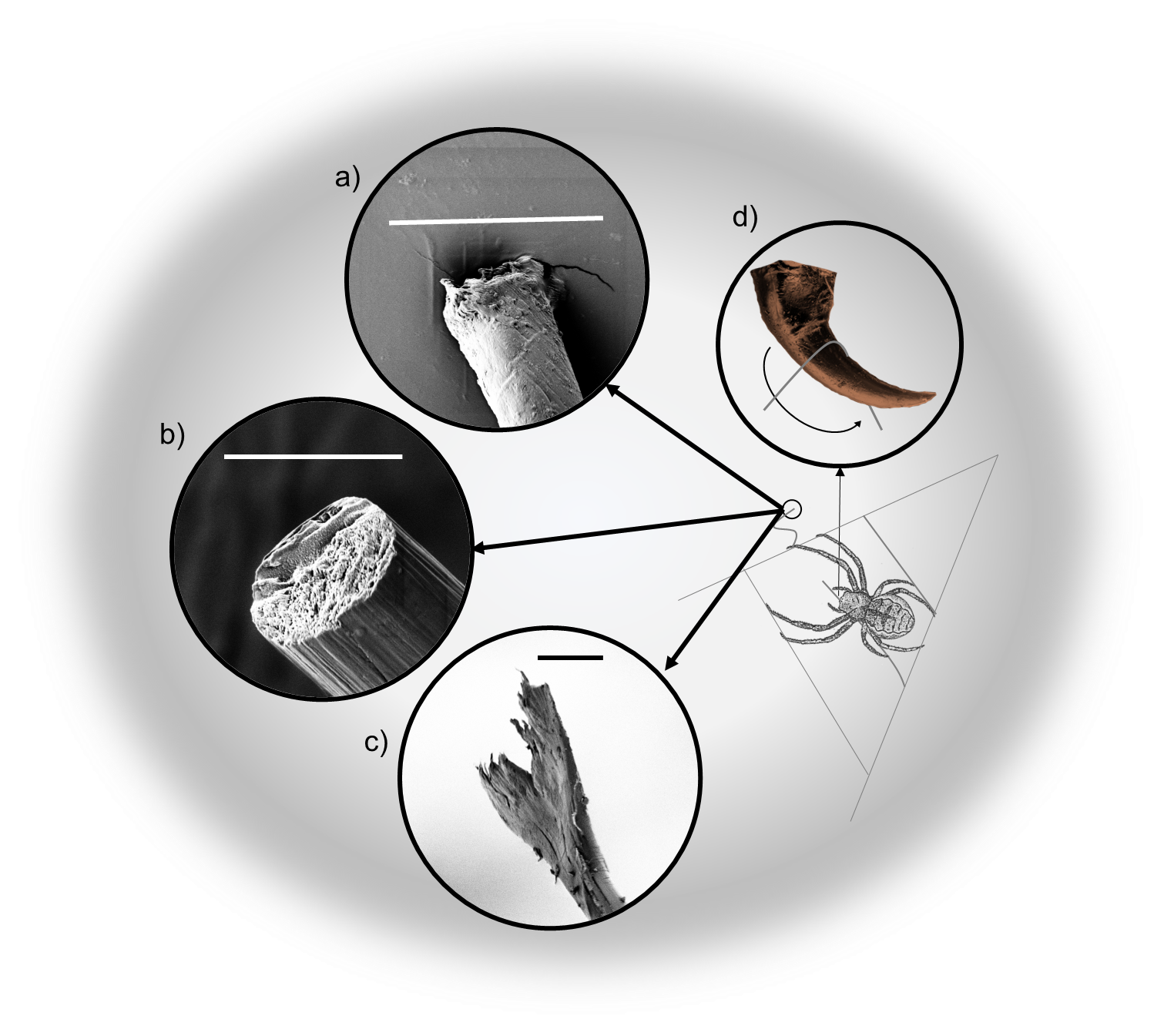


***Fig. S4. Cross-sections of the silk, carbon and Kevlar^®^ fibres cut by spiders.*** *The interaction of the spider and its webs (artificial and natural) has revealed that these animals can cut a) silk lines, b) carbon fibres, and c) Kevlar^®^ fibres (SEM images of the fibres’ cross-sections after the spider cutting). Scale bars 6 μm. d) Hypothesized movement of the fangs to cut the fibres.*


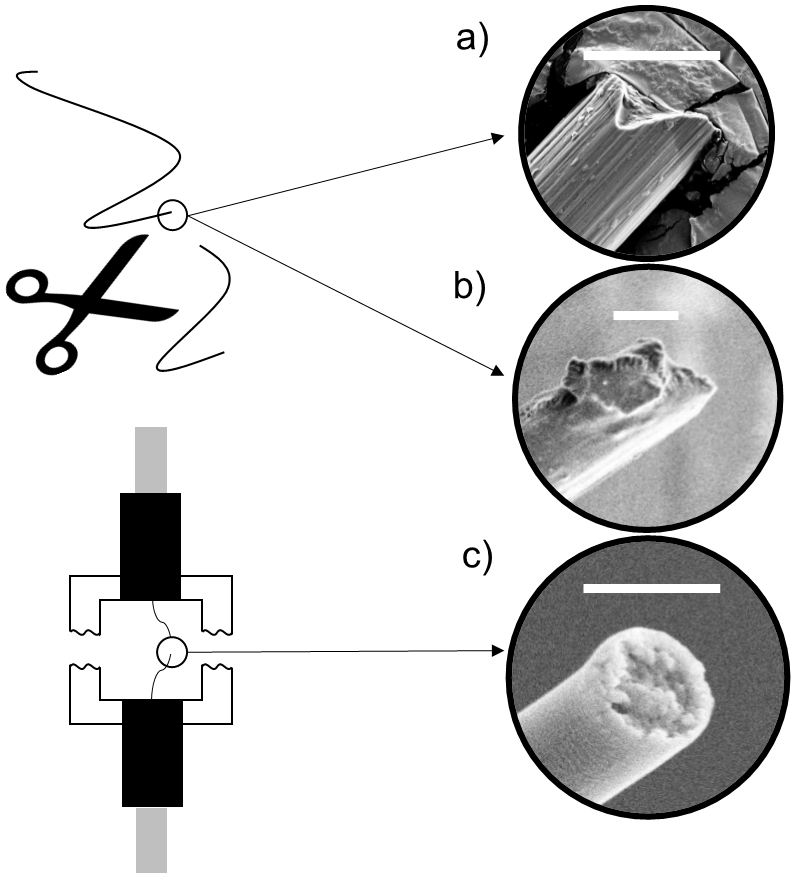


**Fig. S5. The cross-sections of the fibres cut by scissors or tensile tester.** Typical cross sections SEM images of the fibres analysed in this work broken using a pair of scissors and the tensile tester. a) Carbon fibre, b) Kevlar^®^ fibre, and c) spider silk fibre. Scale bars are 6 μm.


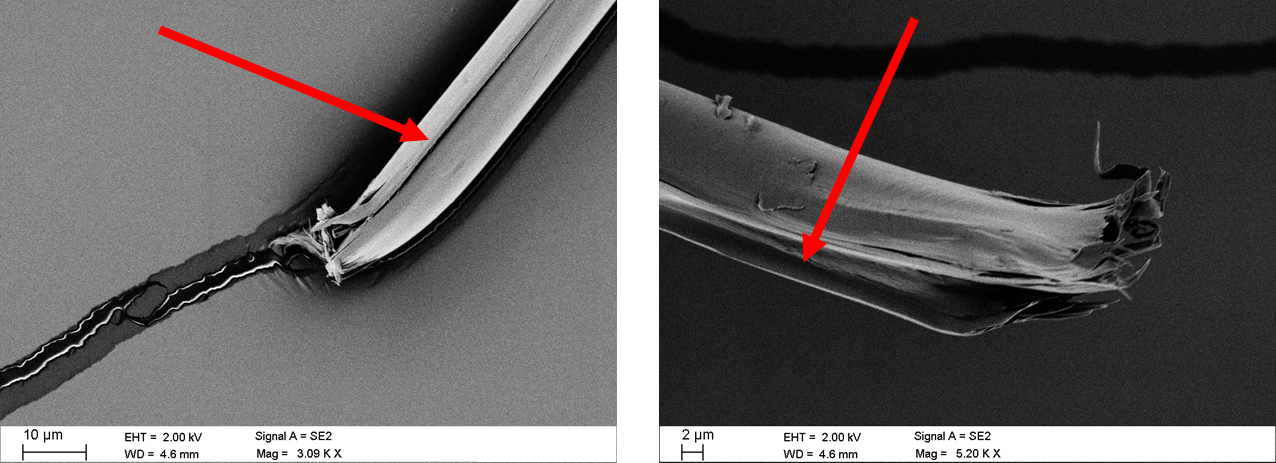


**Fig. S6. The exhausted cross-section of Kevlar^®^ cut by spiders.** Examples of Kevlar^®^ fibres in which it is possible to notice the exhausted and damaged part, here pointed by the red arrow. In this case, the observed cutting was not fragile.


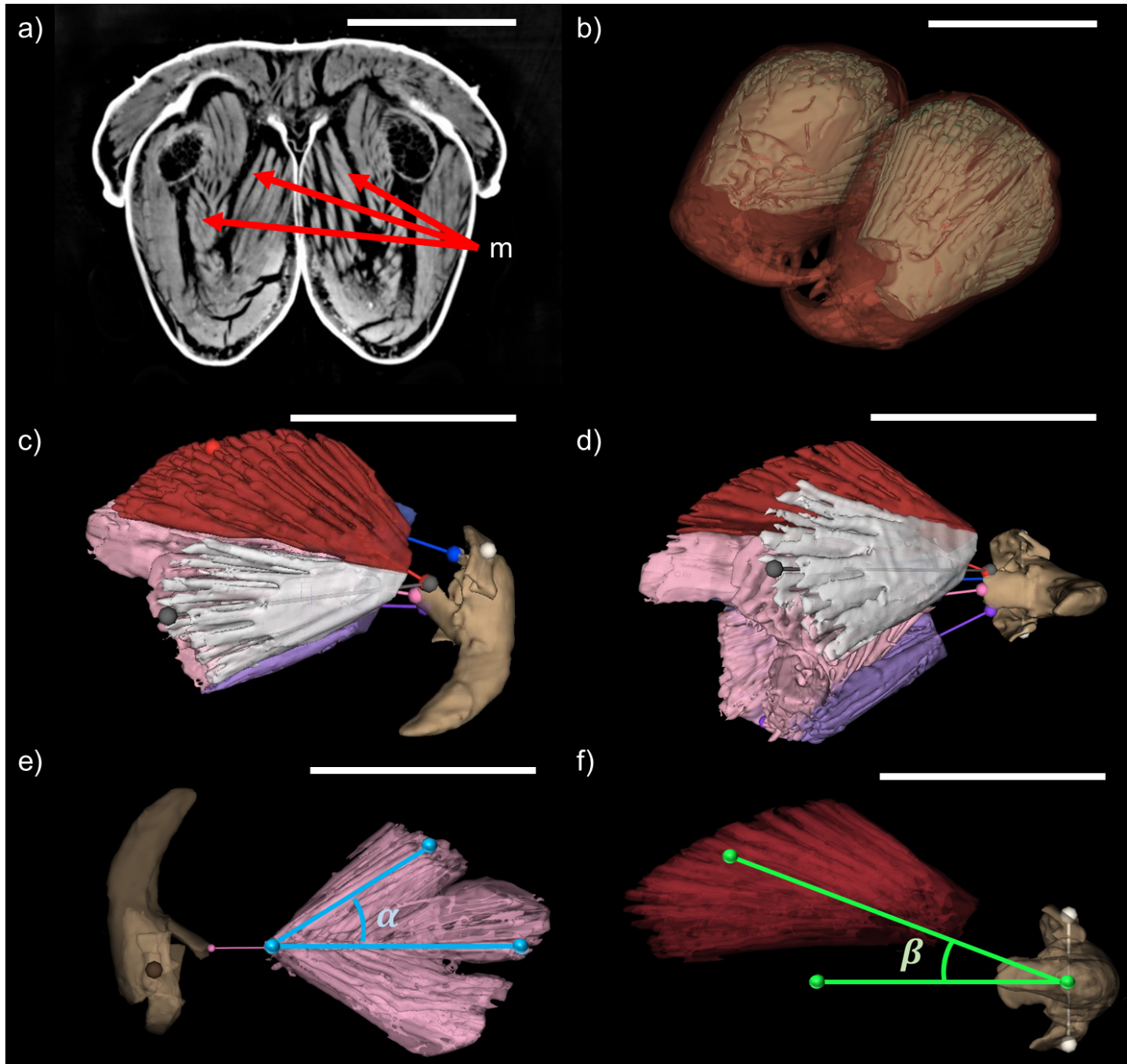


**Fig. S7. Tomography of the muscles in the fang apparatus of spiders.** Micro-tomography reconstructed to show the involved volumes. a) Micro-tomography section of the spider head from which the volumes were reconstructed. Here, m indicates the muscle fibres. b) Structure of the Nuctenea umbratica chelicerae, with the exoskeleton in light transparency to show the muscles, c,d) cleaned muscles from which the maximal force was calculated. e) Pinnation angle $\alpha$ of the central pinnate muscle (pink). f) Attack angle $\beta$ of the superior muscle (red). The axis of rotation of the fang (beige) is in brown for e) and white for f). Scale bars 0.4 mm.


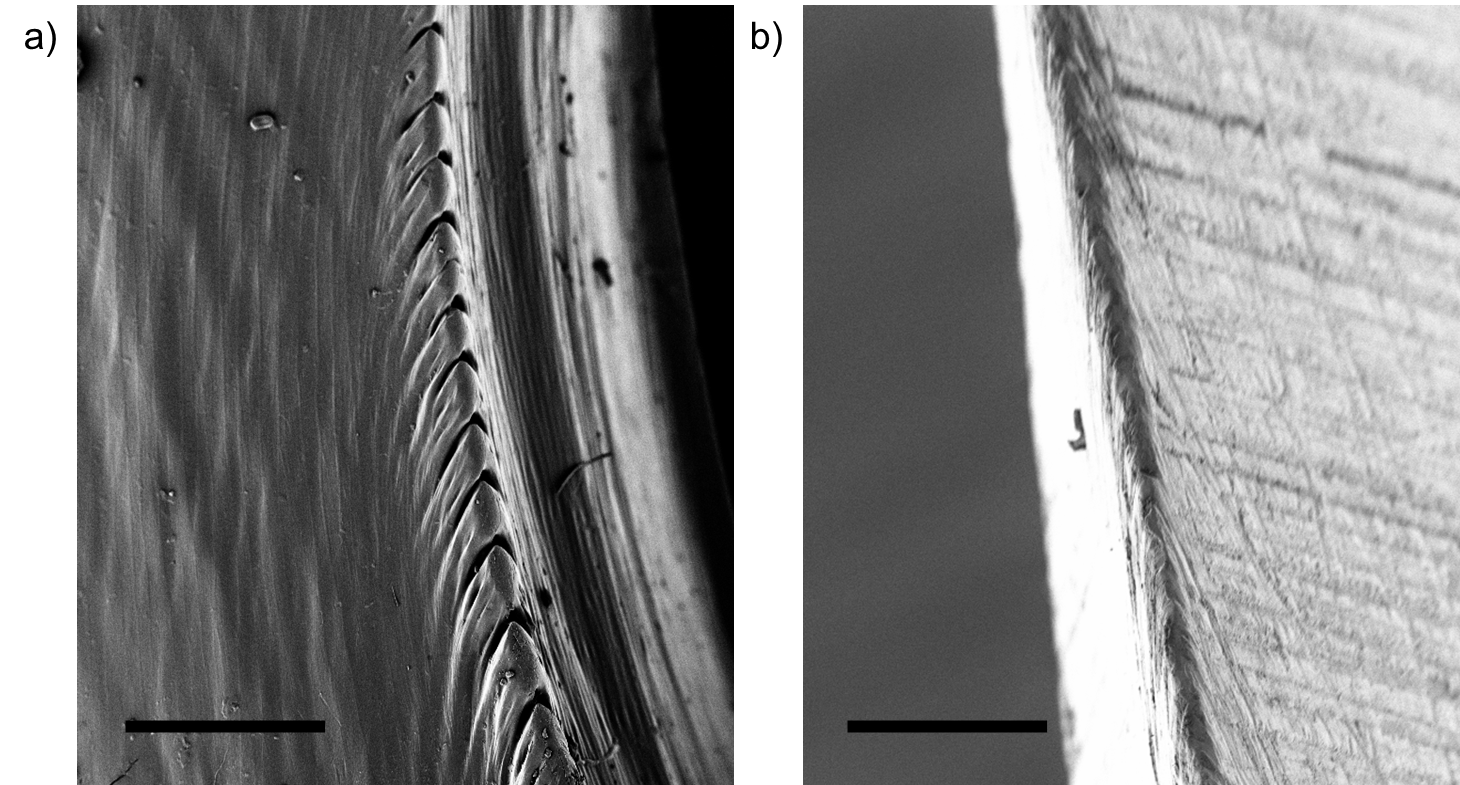


**Fig. S8. Cutting edges with or without serration.** a) Cutting ridge of the spider fang, and b) cutting ridge of the blade #10 used in this work. It is possible to notice the similarity of the edge radius. Scale bars are 50 μm. From these images, it is possible to measure a radius of curvature of about 3.5 μm.


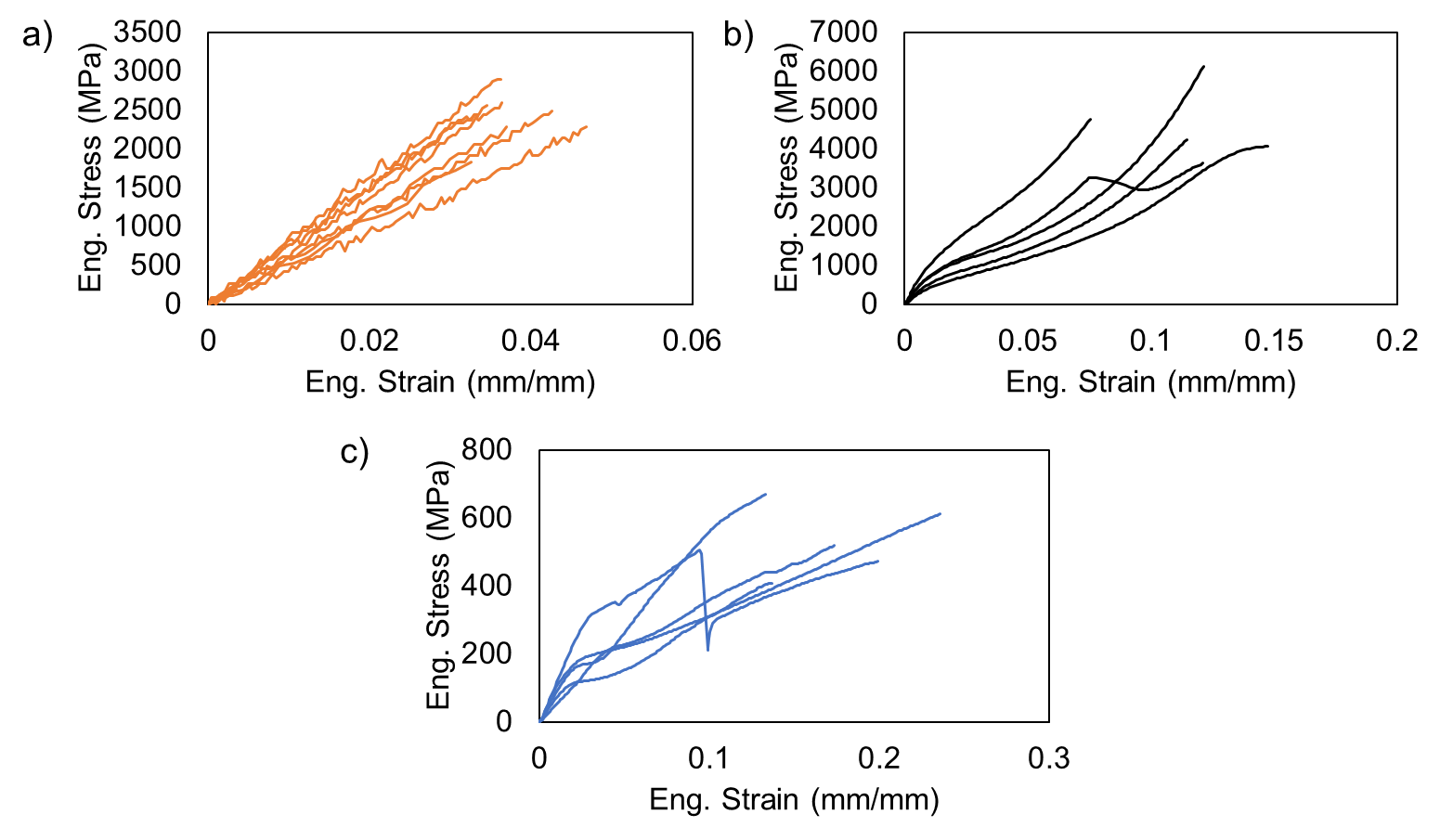


**Fig. S9. Representative stress-strain curves of the fibres that have been tested in this work.** a) Kevlar^®^, b) carbon and c) silk fibres.

**Table S1:** Mechanical properties of the spider major ampullate silk (Nuctenea umbratica) obtained using tensile tests.

| **Major ampullate silk** | **Diameter (μm)** | **Ultimate Strain (mm/mm)** | **Maximal Force (mN)** | **Strength (MPa)** | **Young’s modulus (GPa)** | **Toughness modulus (MJ/m^3^)** |
| --- | --- | --- | --- | --- | --- | --- |
| 1 | 2.8 | 0.22 | 1.82 | 287 | 4.2 | 32 |
| 2 | 2.7 | 0.15 | 2.07 | 366 | 8.8 | 34 |
| 3 | 3.1 | 0.20 | 3.76 | 505 | 12.5 | 72 |
| 4 | 3.1 | 0.33 | 2.58 | 340 | 6.0 | 67 |
| 5 | 4.4 | 0.28 | 2.86 | 188 | 3.4 | 32 |
| 6 | 3.0 | 0.22 | 1.81 | 255 | 5.6 | 32 |
| 7 | 3.1 | 0.23 | 2.64 | 355 | 5.4 | 52 |
| 8 | 3.6 | 0.10 | 1.29 | 130 | 3.4 | 9 |
| 9 | 4.4 | 0.30 | 4.05 | 266 | 7.3 | 46 |
| 10 | 3.1 | 0.18 | 2.88 | 387 | 9.4 | 44 |
| 11 | 4.0 | 0.19 | 3.74 | 301 | 4.8 | 35 |
| 12 | 2.7 | 0.14 | 2.30 | 408 | 7.1 | 30 |
| 13 | 3.5 | 0.34 | 2.78 | 286 | 4.6 | 61 |
| 14 | 2.3 | 0.24 | 2.41 | 586 | 9.9 | 81 |
| 15 | 2.6 | 0.13 | 3.81 | 696 | 8.9 | 51 |
| 16 | 4.0 | 0.10 | 3.33 | 267 | 6.8 | 17 |
| 17 | 3.1 | 0.21 | 3.95 | 519 | 5.8 | 62 |
| **Mean** | **3.3** | **0.20** | **2.83** | **326** | **6.7** | **44** |
| **St. Dev.** | **0.6** | **0.08** | **0.84** | **172** | **2.5** | **20** |

**Table S2:** Mechanical properties of the carbon fibres obtained using tensile tests.

| **Carbon** | **Diameter (μm)** | **Ultimate Strain (mm/mm)** | **Maximal Force (mN)** | **Strength (MPa)** | **Young’s modulus (GPa)** | **Toughness modulus (MJ/m^3^)** |
| --- | --- | --- | --- | --- | --- | --- |
| 1 | 7.1 | 0.05 | 74 | 1870 | 74 | 56 |
| 2 | 7.0 | 0.01 | 189 | 4746 | 108 | 29 |
| 3 | 7.5 | 0.01 | 105 | 2644 | 83 | 32 |
| 4 | 7.4 | 0.03 | 132 | 3329 | 80 | 43 |
| 5 | 7.2 | 0.09 | 111 | 2805 | 63 | 123 |
| 6 | 7.2 | 0.02 | 136 | 3429 | 62 | 38 |
| 7 | 7.2 | 0.02 | 30 | 764 | 95 | 103 |
| 8 | 7.4 | 0.03 | 51 | 1292 | 76 | 26 |
| 9 | 7.1 | 0.04 | 76 | 1905 | 77 | 48 |
| **Mean** | **7.1** | **0.03** | **89** | **2724** | **79** | **55** |
| **St. dev.** | **0.2** | **0.02** | **51** | **1028** | **13** | **34** |

**Table S3:** Mechanical properties of the Kevlar® fibres obtained using tensile tests.

| **Kevlar** | **Diameter (μm)** | **Ultimate Strain (mm/mm)** | **Maximal Force (mN)** | **Strength (MPa)** | **Young’s modulus (GPa)** | **Toughness modulus (MJ/m^3^)** |
| --- | --- | --- | --- | --- | --- | --- |
| 1 | 14.0 | 0.03 | 282 | 1832 | 57 | 31 |
| 2 | 14.3 | 0.04 | 367 | 2286 | 63 | 42 |
| 3 | 13.3 | 0.04 | 345 | 2483 | 56 | 54 |
| 4 | 13.0 | 0.04 | 386 | 2897 | 87 | 54 |
| 5 | 13.2 | 0.06 | 315 | 2293 | 50 | 66 |
| 6 | 13.8 | 0.07 | 312 | 2096 | 64 | 72 |
| 7 | 13.5 | 0.02 | 207 | 1445 | 64 | 15 |
| 8 | 13.8 | 0.03 | 360 | 2408 | 73 | 42 |
| 9 | 12.6 | 0.03 | 272 | 2178 | 71 | 35 |
| 10 | 13.7 | 0.03 | 269 | 1832 | 73 | 24 |
| 11 | 13.7 | 0.03 | 377 | 2558 | 73 | 46 |
| 12 | 14.4 | 0.04 | 385 | 2368 | 73 | 44 |
| 13 | 14.2 | 0.04 | 411 | 2598 | 69 | 47 |
| 14 | 14.5 | 0.05 | 379 | 2286 | 45 | 52 |
| 15 | 13.4 | 0.03 | 292 | 2063 | 71 | 29 |
| 16 | 13.2 | 0.03 | 285 | 2096 | 70 | 35 |
| 17 | 13.5 | 0.03 | 310 | 2178 | 70 | 32 |
| 18 | 13.8 | 0.03 | 281 | 1872 | 63 | 25 |
| 19 | 14.2 | 0.02 | 229 | 1452 | 73 | 15 |
| **Mean** | **13.7** | **0.04** | **319** | **2169** | **67** | **40** |
| **St. Dev.** | **0.5** | **0.01** | **57** | **371** | **9** | **16** |


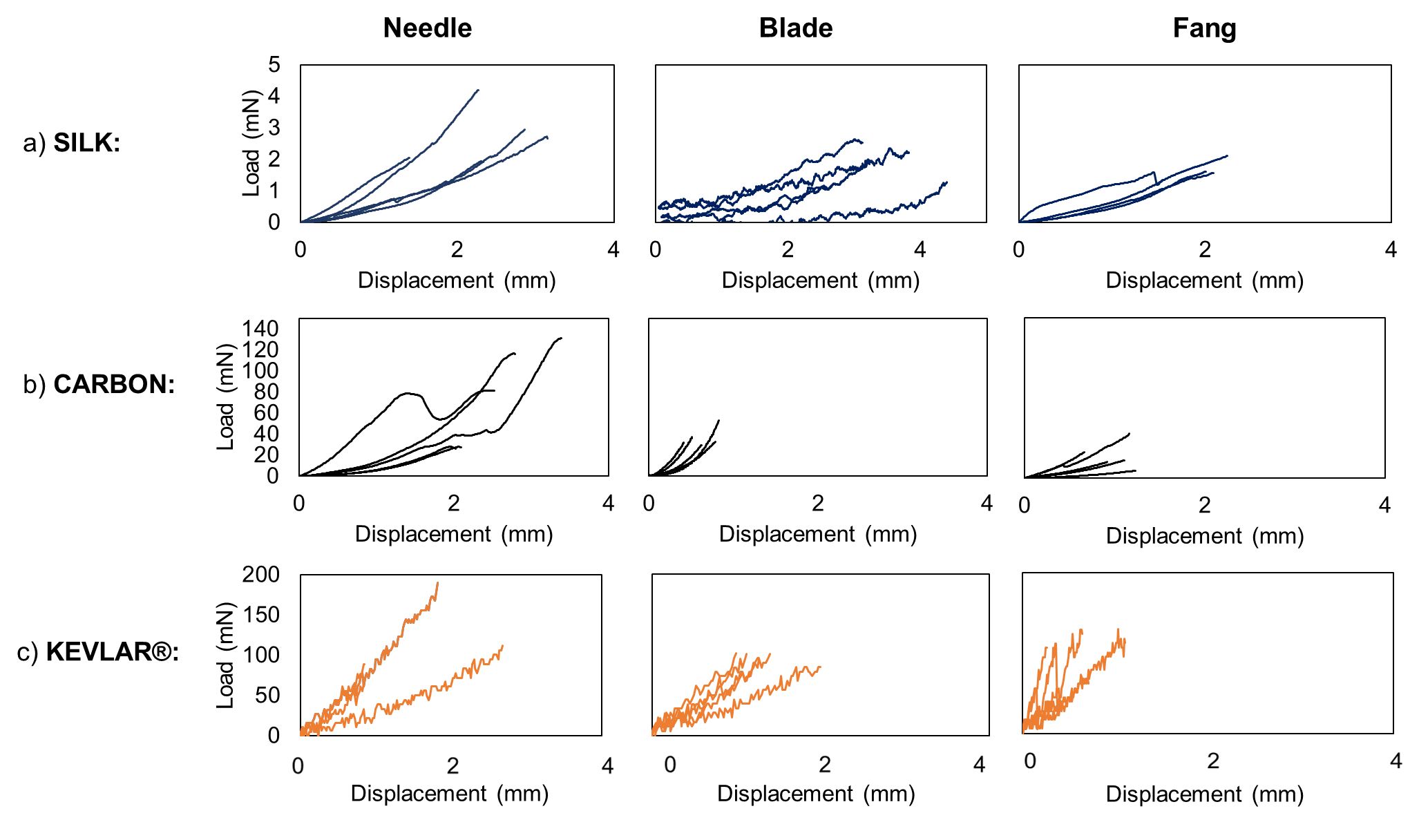


**Fig. S10: Representative load-displacement curves of the fibres that have been tested in the “cutting experiments”**. a) spider silk, b) carbon fibres and c) Kevlar®.

**Table S4:** Mechanical values obtained by breaking the major ampullate silk using the needle.

| **Major ampullate silk (needle)** | **Diameter (μm)** | **Maximal Displacement (mm)** | **Theta (°)** | **Maximal Force (mN)** | **Strength (MPa)** |
| --- | --- | --- | --- | --- | --- |
| 1 | 4.0 | 2.6 | 28 | 1.5 | 132 |
| 2 | 2.1 | 3.6 | 36 | 3.4 | 805 |
| 3 | 2.6 | 2.3 | 25 | 2.1 | 443 |
| 4 | 3.5 | 1.9 | 21 | 0.2 | 35 |
| 5 | 3.5 | 3.8 | 37 | 0.3 | 22 |
| 6 | 3.1 | 2.1 | 23 | 1.8 | 307 |
| 7 | 4.8 | 3.1 | 32 | 2.7 | 136 |
| 8 | 2.6 | 5.7 | 49 | 4.0 | 484 |
| 9 | 4.9 | 4.7 | 43 | 2.0 | 79 |
| 10 | 3.3 | 3.4 | 34 | 1.2 | 130 |
| 11 | 4.0 | 4.3 | 41 | 3.5 | 214 |
| 12 | 4.0 | 2.3 | 25 | 3.2 | 312 |
| 13 | 4.0 | 1.4 | 16 | 2.1 | 308 |
| 14 | 4.0 | 4.5 | 42 | 3.7 | 219 |
| 15 | 3.3 | 3.1 | 32 | 2.7 | 287 |
| 16 | 2.5 | 2.1 | 23 | 0.1 | 35 |
| 17 | 3.7 | 2.9 | 30 | 2.7 | 248 |
| 18 | 3.3 | 2.3 | 25 | 1.9 | 271 |
| 19 | 3.1 | 3.1 | 32 | 0.8 | 96 |
| **Mean** | **3.5** | **3.1** | **31** | **2.1** | **240** |
| **St. Dev.** | **0.7** | **1.1** | **9** | **1.2** | **190** |

**Table S5:** Mechanical values obtained by breaking the major ampullate silk using the blade.

| **Major ampullate silk (blade)** | **Diameter (μm)** | **Maximal Displacement (mm)** | **Theta (°)** | **Maximal Force (mN)** | **Strength (MPa)** |
| --- | --- | --- | --- | --- | --- |
| 1 | 2.4 | 4.4 | 41 | 1.3 | 724 |
| 2 | 1.7 | 4.2 | 40 | 1.5 | 550 |
| 3 | 2.3 | 6.6 | 53 | 1.6 | 241 |
| 4 | 2.2 | 6.0 | 50 | 2.4 | 399 |
| 5 | 2.2 | 5.0 | 45 | 2.1 | 377 |
| 6 | 2.3 | 3.6 | 35 | 2.8 | 585 |
| 8 | 2.5 | 5.7 | 49 | 2.2 | 299 |
| 9 | 4.5 | 5.2 | 46 | 2.5 | 110 |
| 10 | 2.6 | 6.5 | 52 | 2.3 | 284 |
| **Mean** | **2.5** | **5.2** | **46** | **2.1** | **397** |
| **St. Dev.** | **0.8** | **1.1** | **6** | **0.5** | **192** |

**Table S6:** Mechanical values obtained by breaking the major ampullate silk using the fang.

| **Major ampullate silk (fang)** | **Diameter (μm)** | **Maximal Displacement (mm)** | **Theta (°)** | **Maximal Force (mN)** | **Strength (MPa)** |
| --- | --- | --- | --- | --- | --- |
| 1 | 3.2 | 4.0 | 39 | 2.3 | 227 |
| 2 | 3.1 | 3.6 | 36 | 2.7 | 312 |
| 3 | 4.0 | 1.9 | 21 | 2.0 | 226 |
| 4 | 2.9 | 1.6 | 18 | 1.1 | 270 |
| 5 | 1.8 | 3.3 | 34 | 0.7 | 270 |
| 6 | 3.1 | 2.0 | 22 | 0.8 | 136 |
| 7 | 2.6 | 1.1 | 12 | 0.1 | 31 |
| 8 | 2.2 | 0.6 | 7 | 0.1 | 81 |
| 9 | 2.6 | 1.6 | 18 | 1.8 | 533 |
| 10 | 2.6 | 1.5 | 17 | 1.3 | 414 |
| 11 | 2.6 | 4.1 | 39 | 1.0 | 148 |
| 12 | 2.6 | 3.9 | 38 | 3.1 | 464 |
| 13 | 6.2 | 2.1 | 23 | 1.6 | 67 |
| 14 | 3.1 | 2.2 | 24 | 2.1 | 341 |
| 15 | 3.1 | 4.1 | 39 | 2.9 | 307 |
| 16 | 2.6 | 2.1 | 23 | 0.3 | 63 |
| 17 | 2.6 | 2.5 | 27 | 2.6 | 519 |
| 18 | 7.1 | 0.3 | 3 | 0.7 | 147 |
| 19 | 3.1 | 2.6 | 28 | 1.7 | 243 |
| 20 | 3.5 | 2.9 | 30 | 0.4 | 41 |
| **Mean** | **3.2** | **2.4** | **25** | **1.5** | **242** |
| **St. Dev.** | **1.3** | **1.2** | **11** | **1.0** | **156** |

**Table S7:** Mechanical values obtained by breaking carbon fibres using the needle.

| **Carbon fibres (needle)** | **Diameter (μm)** | **Maximal Displacement (mm)** | **Theta (°)** | **Maximal Force (mN)** | **Strength (MPa)** |
| --- | --- | --- | --- | --- | --- |
| 1 | 7.1 | 1.2 | 25 | 65.2 | 1949 |
| 2 | 7.3 | 4.5 | 61 | 52.4 | 754 |
| 3 | 7.1 | 1.7 | 34 | 49.1 | 1097 |
| 4 | 6.9 | 1.0 | 23 | 27.2 | 887 |
| 5 | 7.2 | 1.5 | 31 | 51.8 | 1275 |
| 6 | 7.1 | 5.0 | 64 | 50.6 | 712 |
| 7 | 7.1 | 1.7 | 34 | 42.4 | 954 |
| 8 | 7.2 | 3.5 | 54 | 32.0 | 496 |
| 9 | 7.0 | 1.2 | 26 | 51.2 | 1494 |
| 10 | 7.2 | 2.2 | 41 | 58.2 | 1107 |
| 11 | 6.9 | 1.3 | 27 | 99.1 | 2773 |
| 12 | 6.9 | 1.5 | 31 | 39.2 | 960 |
| 13 | 7.6 | 1.0 | 22 | 26.3 | 883 |
| **Mean** | **7.1** | **2.1** | **36** | **49.6** | **1180** |
| **St. Dev.** | **0.1** | **1.4** | **14** | **18.9** | **605** |

**Table S8:** Mechanical values obtained by breaking carbon fibres using the blade.

| **Carbon fibres (blade)** | **Diameter (μm)** | **Maximal Displacement (mm)** | **Theta (°)** | **Maximal Force (mN)** | **Strength (MPa)** |
| --- | --- | --- | --- | --- | --- |
| 1 | 7.1 | 1.1 | 16 | 53.1 | 2495 |
| 2 | 7.3 | 0.8 | 11 | 29.1 | 1863 |
| 3 | 7.2 | 1.7 | 24 | 37.0 | 1153 |
| 4 | 7.2 | 1.0 | 14 | 40.6 | 2095 |
| 5 | 6.8 | 0.9 | 13 | 31.3 | 1689 |
| 6 | 7.3 | 0.8 | 12 | 31.9 | 2000 |
| 7 | 7.1 | 0.7 | 10 | 31.9 | 2327 |
| 8 | 6.9 | 0.9 | 13 | 41.0 | 2264 |
| 9 | 7.1 | 1.1 | 16 | 32.9 | 1535 |
| **Mean** | **7.1** | **1.0** | **14** | **36.5** | **1936** |
| **St. Dev.** | **0.2** | **0.3** | **4** | **7.5** | **425** |

**Table S9:** Mechanical values obtained by breaking carbon fibres using the fang.

| **Carbon fibres (fang)** | **Diameter (μm)** | **Maximal Displacement (mm)** | **Theta (°)** | **Maximal Force (mN)** | **Strength (MPa)** |
| --- | --- | --- | --- | --- | --- |
| 1 | 7.1 | 0.6 | 13 | 21.5 | 1224 |
| 2 | 7.0 | 0.9 | 20 | 21.3 | 815 |
| 3 | 7.1 | 0.9 | 20 | 20.6 | 784 |
| 4 | 7.3 | 0.6 | 14 | 20.3 | 1005 |
| 5 | 7.2 | 0.9 | 19 | 23.7 | 898 |
| 6 | 7.0 | 0.9 | 19 | 21.7 | 870 |
| 7 | 7.4 | 0.6 | 13 | 12.4 | 617 |
| 8 | 6.8 | 0.8 | 18 | 16.3 | 734 |
| 9 | 7.2 | 0.6 | 12 | 16.4 | 929 |
| 10 | 7.0 | 0.6 | 14 | 6.9 | 377 |
| 11 | 7.3 | 0.8 | 17 | 18.9 | 772 |
| 12 | 7.0 | 1.0 | 22 | 18.1 | 1394 |
| 13 | 7.0 | 0.8 | 17 | 18.3 | 789 |
| 14 | 7.2 | 0.6 | 13 | 19.1 | 2236 |
| 15 | 7.1 | 1.0 | 21 | 16.1 | 1264 |
| 16 | 7.2 | 0.3 | 8 | 19.0 | 2270 |
| 17 | 6.9 | 0.8 | 19 | 18.5 | 1415 |
| **Mean** | **7.1** | **0.7** | **16** | **18.2** | **1082** |
| **St. Dev.** | **0.2** | **0.2** | **4** | **3.9** | **518** |

**Table S10:** Mechanical values obtained by breaking the Kevlar^®^ using the needle.

| **Kevlar^®^ (needle)** | **Diameter (μm)** | **Maximal Displacement (mm)** | **Theta (°)** | **Maximal Force (N)** | **Strength (MPa)** |
| --- | --- | --- | --- | --- | --- |
| 1 | 14.0 | 3.1 | 32 | 0.15 | 930 |
| 2 | 13.3 | 1.7 | 19 | 0.20 | 2202 |
| 3 | 14.2 | 3.3 | 33 | 0.06 | 352 |
| 4 | 14.2 | 1.3 | 15 | 0.09 | 1113 |
| 5 | 13.2 | 1.5 | 17 | 0.16 | 1962 |
| 6 | 13.3 | 1.3 | 14 | 0.15 | 2158 |
| 7 | 12.7 | 1.5 | 16 | 0.19 | 2674 |
| 8 | 14.5 | 2.6 | 28 | 0.11 | 726 |
| 9 | 14.1 | 2.5 | 27 | 0.07 | 474 |
| 10 | 14.1 | 3.0 | 31 | 0.12 | 778 |
| 11 | 14.0 | 2.0 | 22 | 0.15 | 1260 |
| 12 | 13.5 | 2.3 | 24 | 0.21 | 1766 |
| 13 | 13.4 | 1.5 | 17 | 0.18 | 2136 |
| 14 | 14.0 | 1.4 | 16 | 0.15 | 1779 |
| 15 | 14.2 | 1.7 | 19 | 0.17 | 1609 |
| **Mean** | **13.8** | **2.1** | **22** | **0.14** | **1461** |
| **St. Dev.** | **0.5** | **0.7** | **7** | **0.05** | **711** |

**Table S11:** Mechanical values obtained by breaking the Kevlar^®^ using the blade.

| **Kevlar^®^ (blade)** | **Diameter (μm)** | **Maximal Displacement (mm)** | **Theta (°)** | **Maximal Force (N)** | **Strength (MPa)** |
| --- | --- | --- | --- | --- | --- |
| 1 | 13.2 | 1.9 | 21 | 0.10 | 961 |
| 2 | 13.5 | 2.0 | 22 | 0.15 | 1333 |
| 3 | 13.8 | 1.1 | 12 | 0.08 | 1286 |
| 4 | 13.5 | 1.5 | 16 | 0.09 | 1029 |
| 5 | 13.6 | 1.3 | 15 | 0.11 | 1490 |
| 6 | 13.3 | 1.7 | 19 | 0.08 | 877 |
| 7 | 13.4 | 2.3 | 25 | 0.10 | 827 |
| 8 | 13.6 | 1.5 | 16 | 0.10 | 1224 |
| 9 | 14.4 | 1.0 | 12 | 0.12 | 1987 |
| 10 | 14.2 | 2.6 | 27 | 0.09 | 633 |
| 11 | 12.9 | 1.1 | 12 | 0.10 | 1652 |
| 12 | 14.5 | 2.4 | 25 | 0.10 | 761 |
| 13 | 13.6 | 2.3 | 25 | 0.08 | 676 |
| 14 | 13.6 | 1.9 | 21 | 0.08 | 804 |
| **Mean** | **13.6** | **1.7** | **19** | **0.10** | **1110** |
| **St. Dev.** | **0.5** | **0.5** | **5** | **0.02** | **402** |

**Table S12:** Mechanical values obtained by breaking the Kevlar^®^ using the fang.

| **Kevlar^®^ (fang)** | **Diameter (μm)** | **Maximal Displacement (mm)** | **Theta (°)** | **Maximal Force (N)** | **Strength (MPa)** |
| --- | --- | --- | --- | --- | --- |
| 1 | 13.2 | 0.7 | 7 | 0.09 | 2533 |
| 2 | 13.5 | 0.5 | 6 | 0.08 | 2509 |
| 3 | 13.4 | 0.5 | 6 | 0.09 | 3117 |
| 4 | 13.4 | 0.3 | 3 | 0.07 | 3794 |
| 5 | 13.1 | 0.6 | 7 | 0.07 | 2099 |
| 6 | 13.5 | 0.6 | 7 | 0.11 | 3128 |
| 7 | 14.5 | 0.7 | 8 | 0.09 | 2220 |
| 8 | 13.9 | 0.5 | 6 | 0.06 | 1787 |
| 9 | 14.3 | 0.5 | 6 | 0.07 | 2318 |
| 10 | 12.8 | 0.6 | 7 | 0.11 | 2997 |
| 11 | 13.6 | 0.6 | 7 | 0.10 | 2778 |
| 12 | 14.3 | 0.4 | 5 | 0.07 | 2592 |
| 13 | 14.2 | 0.6 | 7 | 0.08 | 2185 |
| 14 | 12.8 | 0.9 | 10 | 0.11 | 2141 |
| 15 | 13.8 | 3.1 | 32 | 0.04 | 259 |
| 16 | 13.7 | 2.5 | 27 | 0.06 | 425 |
| 17 | 13.9 | 1.8 | 20 | 0.08 | 780 |
| 18 | 13.2 | 2.8 | 30 | 0.06 | 385 |
| 19 | 13.3 | 1.5 | 17 | 0.11 | 1265 |
| 20 | 14.6 | 4.4 | 41 | 0.06 | 320 |
| 21 | 14.0 | 3.1 | 32 | 0.11 | 683 |
| 22 | 14.2 | 5.8 | 49 | 0.12 | 555 |
| **Mean** | **13.7** | **1.5** | **14** | **0.08** | **1858** |
| **St. Dev.** | **0.5** | **1.5** | **1** | **0.02** | **1088** |


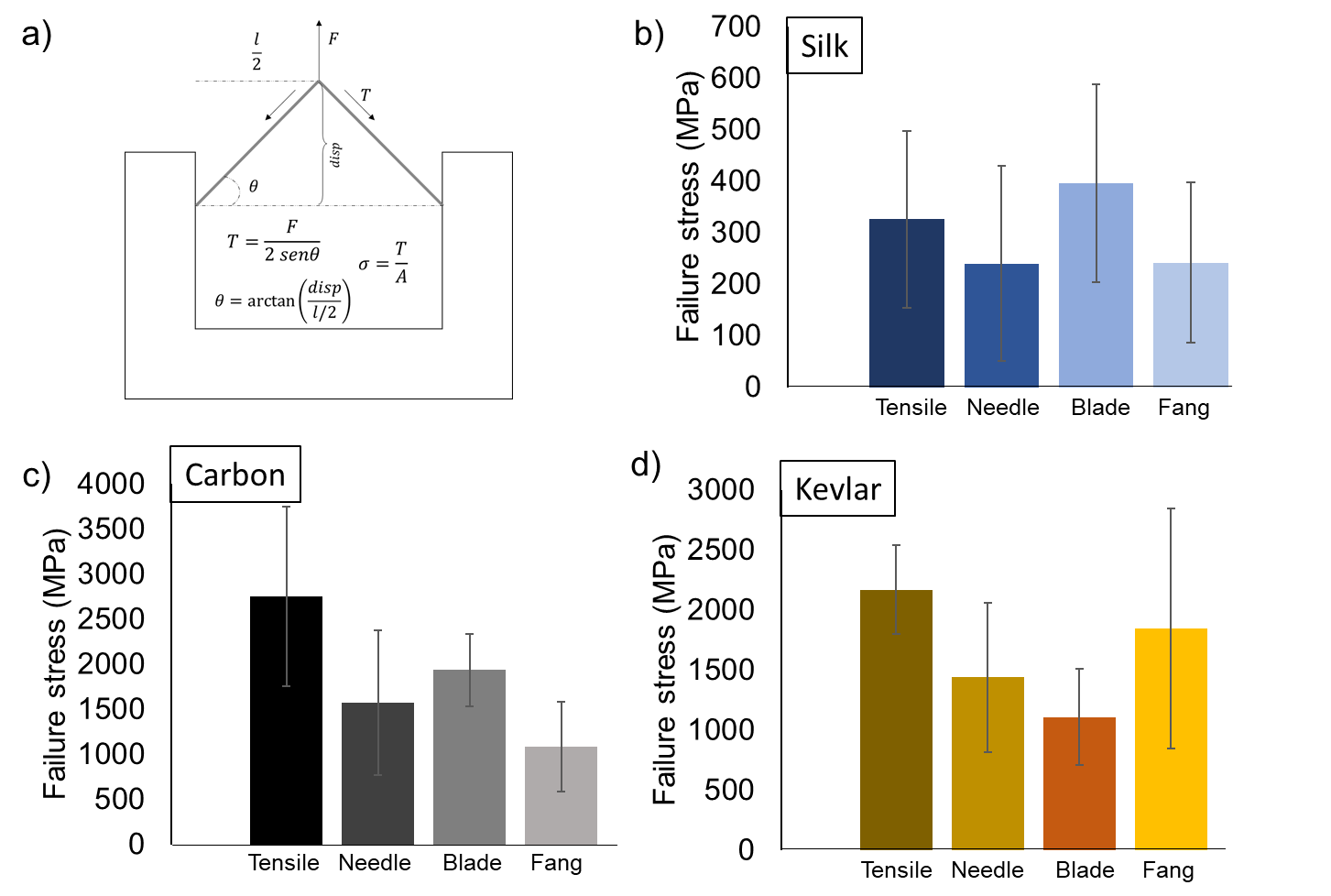


**Fig. S11. The failure stress calculation in the micromechanical cutting experiments.** a) Schematic of the model used to calculate the tension in the fibre from the force recorded in the customized micromechanical setup. Values of the strength obtained for the different types of fibres with the different setups, b) major ampullate silk, c) carbon fibres, and d) Kevlar^®^ fibres. The sample size for each experiment was between 9 to 22 and the analysis was performed using Excel^®^.


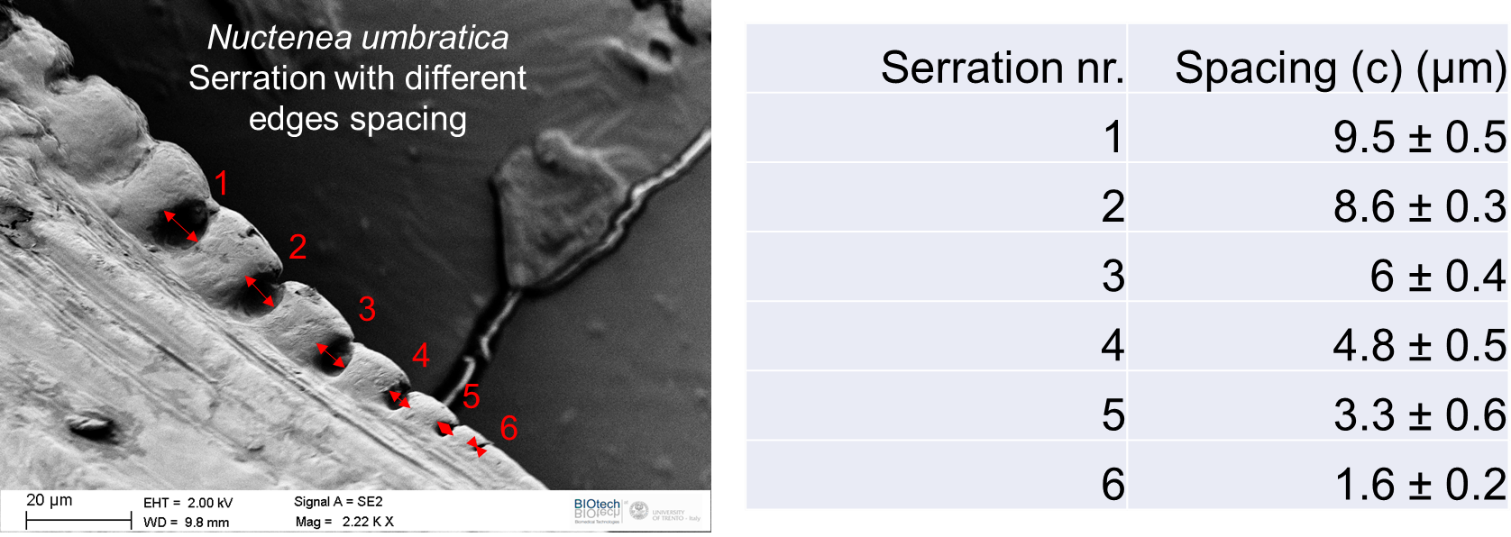


**Fig. S12. The non-uniform spacing of the serration in spiders.** Measurements of the spacing (c) between serration in the species Nuctenea umbratica. It is possible to see that, contrary to what happens for dinosaurs and sharks, the serration here presented is graded in morphology.


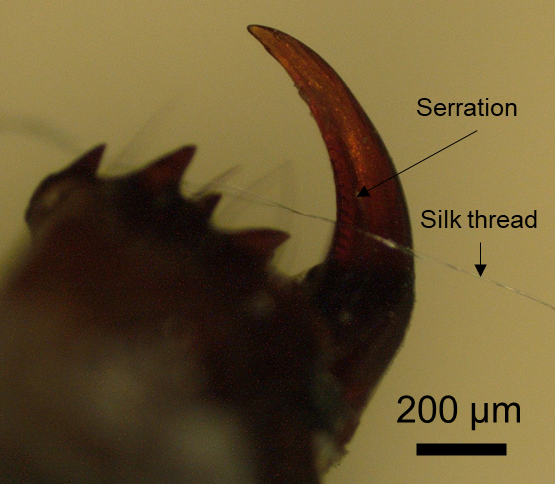


**Fig. S13. Fang of a Nuctenea umbratica with the relative silk thread for comparison of the dimensions of the serration and the fibre.**

**Section S1: Estimation of the mechanical force generated by the chelicerae muscles**

To investigate the biomechanics of the fang and estimate the maximum force sustainable by the muscles of the fang in the closed position while the paws pull, we performed 3D μ-tomography. With the ImageJ plug-in “WEKA trainable segmentation”, we classified the grey-scale images into *exoskeleton*, *muscles* and *background* classes. We then built a 3D model of the exoskeleton and muscles with the segmented images in the software 3D-Slicer.

There is no separation between the fang and exoskeleton; they are connected through two flexible thickenings of the shell which determine its axis of rotation (Figure S6). We identified five muscles, four flexors (white, red, violet and pink) and one extensor (blue) (see Supplementary Video 4). The tendons are anchored to the protrusions at the base of the fang. The flexors are anchored to the large medial protrusion while the extensor is anchored to the small lateral and central protrusions. The 3D reconstruction allowed us to measure the mean physiological cross-sectional area ($A_{cross}$) of the muscle bundles, the moment arms that the respective tendons have in reference to the axis of rotation, pennation and attack angles, and thus, moments (Table SS1). The specific tension $T$ of a spider muscle is in the order of 1000 kPa(*27*, *28*). Each muscle has a certain resultant force $F_{res}$ that generates a momentum $M=F_{res}\cdot D$ (Eq.1) with respect to the axis of rotation, with $D$ the arm between the tendon and the axis of rotation on the fang. We calculated $F_{res}$ as $T\cdot A_{cross}\cdot cos(\alpha)\cdot cos(\beta)$*,* (Eq.2) where $\alpha$ is the muscle pennation angle (Figure S6e) and $\beta$ the angle between the tendon and the plane perpendicular to the axis of rotation (Figure S6f). The total momentum that the muscles can exert in the closed isometric contraction is$M_{tot}\cong32.55\cdot{10}^{-7} \mathrm{Nm}$. The force with which the silk thread is pulled that can be sustained by the muscles is $M_{tot}/r$, with $r$ the distance from the axis of rotation of the serration on which the fibre is positioned. Having the serration ends $r$ =120 and 190 $\mu m$, the maximum and minimum $F$ are 27.13 and 17.13 mN, respectively.

*Table S13. Mean cross-section area (*$A_{cross}$*) of the muscle bundles, moment arms (*$d$*), pennation (*$\alpha$*) and attack (*$\beta$*) angles measured on the 3D model, and moments. The color in parenthesis is referred to Figure 4.*

| *Muscle* | $A_{cross} [{10}^{6}{\mu m}^{2}]$ | $\alpha$ *[°]* | $\beta$ *[°]* | $D$ $[\mu m]$ | $M$ *[*${10}^{-7}$Nm*]* |
| --- | --- | --- | --- | --- | --- |
| *Superior (red)* | *0.02041* | *10* | *20* | *47* | *8.8781* |
| *Medial (white)* | *0.00838* | *0* | *0* | *0* | *0* |
| *Central Pinnate (pink)* | *0.05103* | *24.5* | *0* | *42* | *19.4873* |
| *Lateral (violet)* | *0.01348* | *0* | *20* | *33* | *4.1821* |


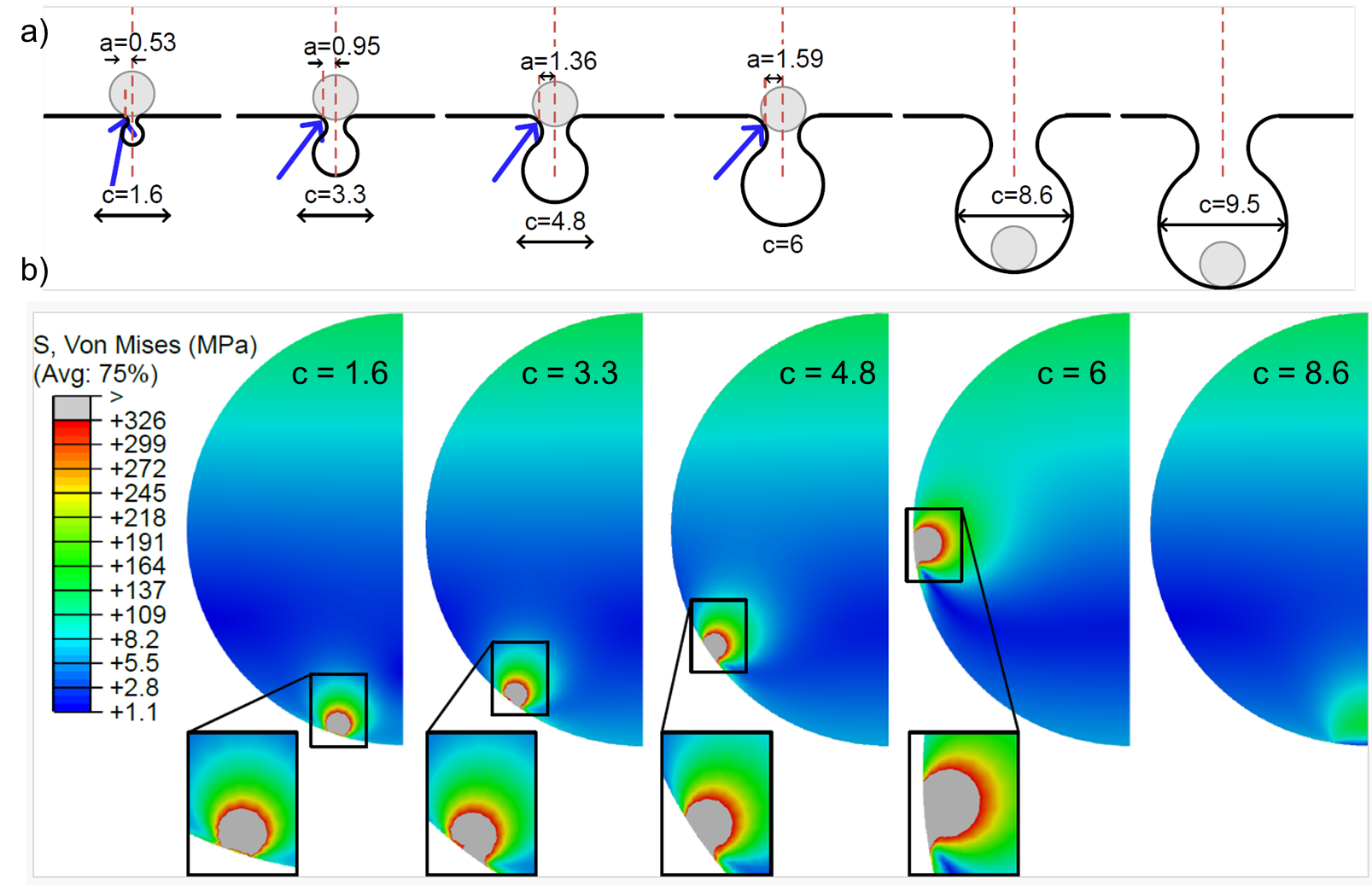


**F*igure S14: Estimation of the interaction between the serration and the silk fibres.*** *a) The schemes reported in the figure have been obtained from the 3D serrations modelled in SolidWorks and used in the Abaqus simulations. In the figure are highlighted the crack lengths a (defects) as a function of the serrated edges c. Such crack lengths (a). b) von-Mises stresses,* $\sigma_{VM}$*, within the silk fibres are depicted for various serrated edges c: (a) 1.6 mm, (b) 3.292 mm, (c) 4.782 mm, (d) 6.012 mm, and (e) 8.643 mm. The stresses were assessed under a uniform transversal displacement of 0.5 mm. Notably, the region with von-Mises stresses surpassing the mean tensile strength of 326 MPa is more pronounced for c=6, corresponding to the highest a/R ratio.*

**Table S14:** Results of the simulations concerning the load required to achieve an Area in the fibre of 0.024 µm^2^ where the von-Mises stress is higher than 326 MPa, which is the experimentally obtained strength of the silk.

| Serration dimension, *c* [μm] | Major ampullate silk | | | |
| --- | --- | --- | --- | --- |
|  | *a* [μm] | Area with  von-Mises stress  >326 MPa [µm^2^] | *a/R* | Force to cut reduction  compared to absence of serration (%) |
| 1.6 | 0.53 | 0.024 | 0.31 | 75 |
| 3.292 | 0.95 | 0.024 | 0.57 | 75 |
| 4.782 | 1.36 | 0.024 | 0.82 | 76 |
| 6.012 | 1.59 | 0.024 | 0.96 | 80 |
| 8.643 | / | 0.024 | / | 0 |
| 9.514 | / | 0.024 | / | 0 |

**Section S2: Analytical model of the cutting, smart positioning and optimal cutting.**

We assume that the fibre is cut when its compression on the fang generates a stress ($\sigma$) at least equal to its material strength ($\sigma_{c})$:

$$\sigma=\frac{P}{B}=\sigma_{c}$$

where *P* is the compression load on the fibre on the fang and *B* is a characteristic area expected to be close to the cross-sectional area of the fibre *A*. If there is no serration and no pre-tension applied to the fibre, the local contact area (of the order of *B*) between the fang and the fibre can be locally described with the Hertz model (*51*) of two spherical bodies in contact, considering the radius of curvature of the two elements in contact. Assuming that the fang and the fibre have the same Poisson’s ratio ($\nu$) we can thus write:

$$\sigma=Z P^{\frac{1}{3}}$$

with $Z= \frac{3}{2\pi}\left( \frac{3}{4} \right)^{-\frac{2}{3}}\left( 1-\nu^{2} \right)^{-\frac{2}{3}}\left( \frac{1}{E_{1}}+\frac{1}{E_{2}} \right)^{-\frac{2}{3}}\left( \frac{R_{1}R_{2}}{R_{1}+R_{2}} \right)^{-\frac{2}{3}} ,$where *E_1_* and *E_2_* are the two Young’s moduli of the fang and the fibre, and *R_1_* and *R_2_* are their two local radii of curvature.

We can now consider the contact between a fibre and a fang (in which the former is perpendicular to the latter), see Figure 3 in the main manuscript.

Both the presence of the serration on the edge of the fang and of the pre-tension applied by the spider with the legs aid the cutting, meaning that the load necessary to cut the fibre in the absence of serration and/or pre-tension is higher with respect to the one in presence of serration and/or pre-tension. We can thus define a cutting efficiency as:

$$Cutting efficiency=1-\frac{P_{ST}}{P_{0}} (1)$$

where *P_ST_* is the load to cut the fibre with serration (*P_S_* if only with the serration) and a pre-tension (*P_T_* if only with the pre-tension) and *P*_0_ is the critical load necessary to cut the fibre in the absence of serration and pre-tension, here defined as control condition of negligible cutting efficiency.

From equation 1 it can be seen that if the cutting efficiency is positive the cutting is aided, by either the serration or the pre-tension. For example, if cutting efficiency is 0.3 the force to cut the fibre is 30% lower with respect to the control condition. If it is negative, it means that the load required to cut the fibre is higher, meaning that the condition is disadvantageous for cutting. In the following three subsections, we will calculate the cutting efficiency in the presence of serration, pre-tension on the fibre, and both serration and pre-tension.

1. *The effect of the serration:*

To quantify the effect of serration on cutting efficiency we used the schematic depicted in Figure S12 and Figure 4, which highlight the presence of two contact points.


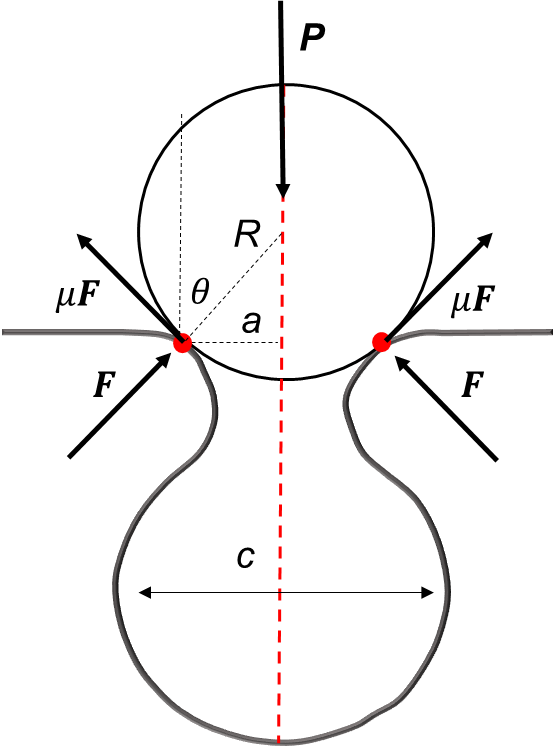


Figure S15: Schematic of the fibre pressed in a serrated edge. The contact points are the red dots.

The fibre (radius *R*) is compressed on the serration with a compressive load *P*. The relevant geometrical parameters are defined as following:

$$a=R\sin\theta\Rightarrow\sin\theta=\frac{a}{R}$$

$$\cos\theta=\sqrt{1-\left( \frac{a}{R} \right)^{2}}$$

where the distance *a* is related to the length of the serration $c\propto a$. $\mu$ is the friction coefficient between the fibre and the fang. From the equilibrium of the vertical components of the forces of reactions, friction forces and applied compressive load we have:

$$2 F\cos\theta+2 \mu F\sin\theta=P$$

From which we can obtain the reaction force *F* of the fang:

$$2 F\left( \sqrt{1-\left( \frac{a}{R} \right)^{2}}+\mu\frac{a}{R} \right)=P$$

The compressive load in the absence of serration and assuming independent contact stress fields is (*P*_0_*_max_*) and can be defined in the case $\frac{a}{R}\to0$ that is:

$$P_{0max}=2 F$$

Note that a factor of 2 appears with respect to the real case of absence of serration, i.e. $P_{0min}=P_{0}= F$ since in reality in the absence of serration the contact point is one and not two independent.

In any case, from this, we can relate the compressive load in case of presence (*P_S_*) or absence of serration ($P_{0}$) as:

$$\frac{P_{s}}{P_{0}}=\sqrt{1-\left( \frac{a}{R} \right)^{2}}+\mu\frac{a}{R}$$

This ratio is ruled by *a/R* and it shows the condition for which the serration is most/least favorable for cutting fibres. For example, the worst condition for cutting (that becomes the best condition in the design of cutting-resistant fibres) can be obtained by imposing:

$$worst cutting\Rightarrow\frac{1}{P_{0}}\frac{dP_{s}}{d\left( \frac{a}{R} \right)}=0$$

$$\frac{1}{2}\left( 1-\left( \frac{a}{R} \right)^{2} \right)^{-\frac{1}{2}}\left( -\frac{2a}{R} \right)+\mu=0\Rightarrow\frac{\frac{a}{R}}{\sqrt{1-\left( \frac{a}{R} \right)^{2}}}=\mu$$

that gives:

$$\left( \frac{a}{R} \right)_{worst}=\frac{\mu}{\sqrt{1+\mu^{2}}}$$

and with $\left( \frac{a}{R} \right)_{worst}$ it is possible to calculate the max ratio between the compressive load in the presence and absence of serration as:

$$\left. \frac{P_{s}}{P_{0}} \right|_{max}=\sqrt{1-\frac{\mu^{2}}{1+\mu^{2}}}+\frac{\mu^{2}}{\sqrt{1+\mu^{2}}}=\sqrt{1+\mu^{2}}$$

The condition necessary to have an optimal cutting due to serration is maximizing $a/R$ (Figure 4a), which gives *a/R* theoretically around 1; note that serration starts to help for values of efficiency>0, e.g. *a/R*>0.54 for $\mu$=0.3 or *a/R*>0.8 for $\mu$=0.5, suggesting that the lower the friction the sooner and the higher is the positive effect of serration. For $\mu$=0.3, and 0.5 the load to break the fibre in the presence of serration is reduced by a factor of 56%, and 36% respectively.

1. *The effect of the pre-tension:*

The stress generated by the contact between the fibre and the fang could not be the only one in play. In fact, the spider may willingly put the fibre under pre-tension using its legs, thus aiding the cutting. In this context, we consider the maximal tension in the fibre using the von-Mises approach, as:

$$\sigma_{M}=\sqrt{\sigma_{H}^{2}+\sigma_{T}^{2}}$$

where $\sigma_{H}$ is the tension obtained with the previously discussed Hertz approach whereas $\sigma_{T}$ is the pre-tension induced by the spider when applying an additional traction force *F_T_*. The last relation can be thus rewritten as:

$$\sigma_{M}=\sqrt{Z^{2}P^{\frac{2}{3}}+\frac{F_{T}^{2}}{A^{2}}}$$

Accordingly, cutting takes place when $\sigma_{M}=\sigma_{c}$ resulting in the following cutting force *P*.

$$\sigma_{C}^{2}=\frac{F^{2}}{A^{2}}+Z^{2}P^{\frac{2}{3}}\Rightarrow P^{\frac{2}{3}}=\frac{\sigma_{c}^{2}-\frac{F_{T}^{2}}{A^{2}}}{Z^{2}}$$

$$\Rightarrow P=\left( \frac{\sigma_{c}^{2}-\frac{F_{T}^{2}}{A^{2}}}{Z^{2}} \right)^{\frac{3}{2}}$$

From this relation, it is possible to see that having a pretension on the fibre drastically reduces the compression load necessary to cut the fibre. In particular, we can calculate the ratio between the load necessary to cut the fibre in the presence or absence (*F_T_=0*) of pre-tension, as

$$\frac{P_{T}}{P_{0}}=\left( \frac{\frac{\frac{\sigma_{C}^{2}-\frac{F_{T}^{2}}{A^{2}}}{Z^{2}}}{\sigma_{c}^{2}}}{Z^{2}} \right)^{\frac{3}{2}}=\left( 1-\frac{F_{T}^{2}}{A^{2}\sigma_{c}^{2}} \right)^{\frac{3}{2}}=\left( 1-\frac{\sigma_{T}^{2}}{\sigma_{c}^{2}} \right)^{\frac{3}{2}}$$

where $\sigma_{c}$ can be easily obtained from the experimental data on tensile tests. From Figure 4b it is possible to see that having a pre-tension on the fibre always positively affects cutting efficiency. In particular, if we consider a load that is half of the one necessary to break the fibre $\left( \frac{\sigma_{T}}{\sigma_{c}}=\frac{1}{2} \right)$, we obtain a cutting efficiency of about 40%.

1. *The effect of the serration and pre-tension:*

In order to evaluate the total cutting efficiency in the presence of both serration and pre-tension simultaneously, we can again apply the von-Mises approach noting that the previous result remains valid, i.e.:

$$\frac{P_{sT}}{P_{s}}=\frac{P_{T}}{P_{0}}=\left( 1-\frac{\sigma_{T}^{2}}{\sigma_{c}^{2}} \right)^{\frac{3}{2}}$$

thus:

$$\frac{P_{sT}}{P_{0}}=\frac{P_{s}}{P_{0}}\frac{P_{T}}{P_{0}}= \left( 1-\frac{\sigma_{T}^{2}}{\sigma_{c}^{2}} \right)^{\frac{3}{2}}\left( \sqrt{1-\left( \frac{a}{R} \right)^{2}}+\mu\frac{a}{R} \right) (2)$$

From figure 4c it is possible to see that having a pre-tension and a serrated edge drastically reduces the load necessary to cut the fibre. A ratio *a/R=*0.84 gives a cutting efficiency of 30% in the absence of pre-tension, which can raise up to 50% by applying a pre-tension of $\frac{\sigma_{T}}{\sigma_{c}}$ = 0.45.

1. *Smart positioning*

A direct consequence of this analytical model is the importance of the smart positioning of the fibre intended to be cut along the serrated edge of the fang, for *a/R* approaching 1. Thanks to the graded serration of spider fangs (i.e. different *c* and thus *a*) the spider is able to smartly reach this optimal positioning for the optimal cutting just by sliding the fibre on the fang in the right direction up to when the fibre will be naturally fixed in the optimal configuration. Figures 4d,e show the proposed smart positioning mechanism. Basically, the fibre slides on the different serrated edges till it gets locked in the one where the cutting load is minimal, as demonstrated by our model.


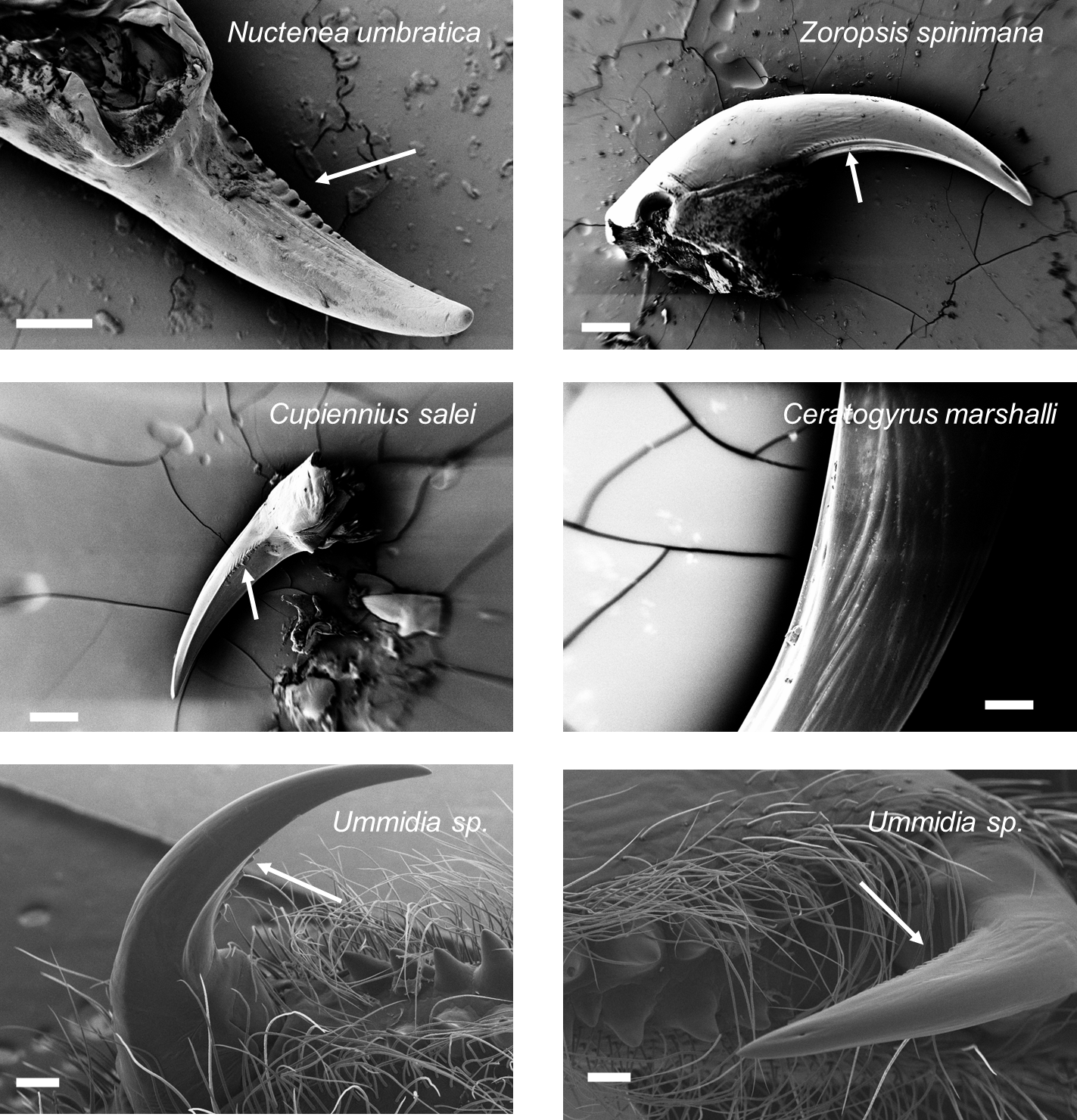


**Figure S16: SEM images of different spiders’ fangs.** It is possible to notice that in the Theraphosidae one, i.e. Ceratogyrus marshalli, no serration is evident as it is for the others. On the other hand, in Ummidia sp. (Mygalomorphae, Halonoproctidae) the serration is evident. Scale bars 200 μm.


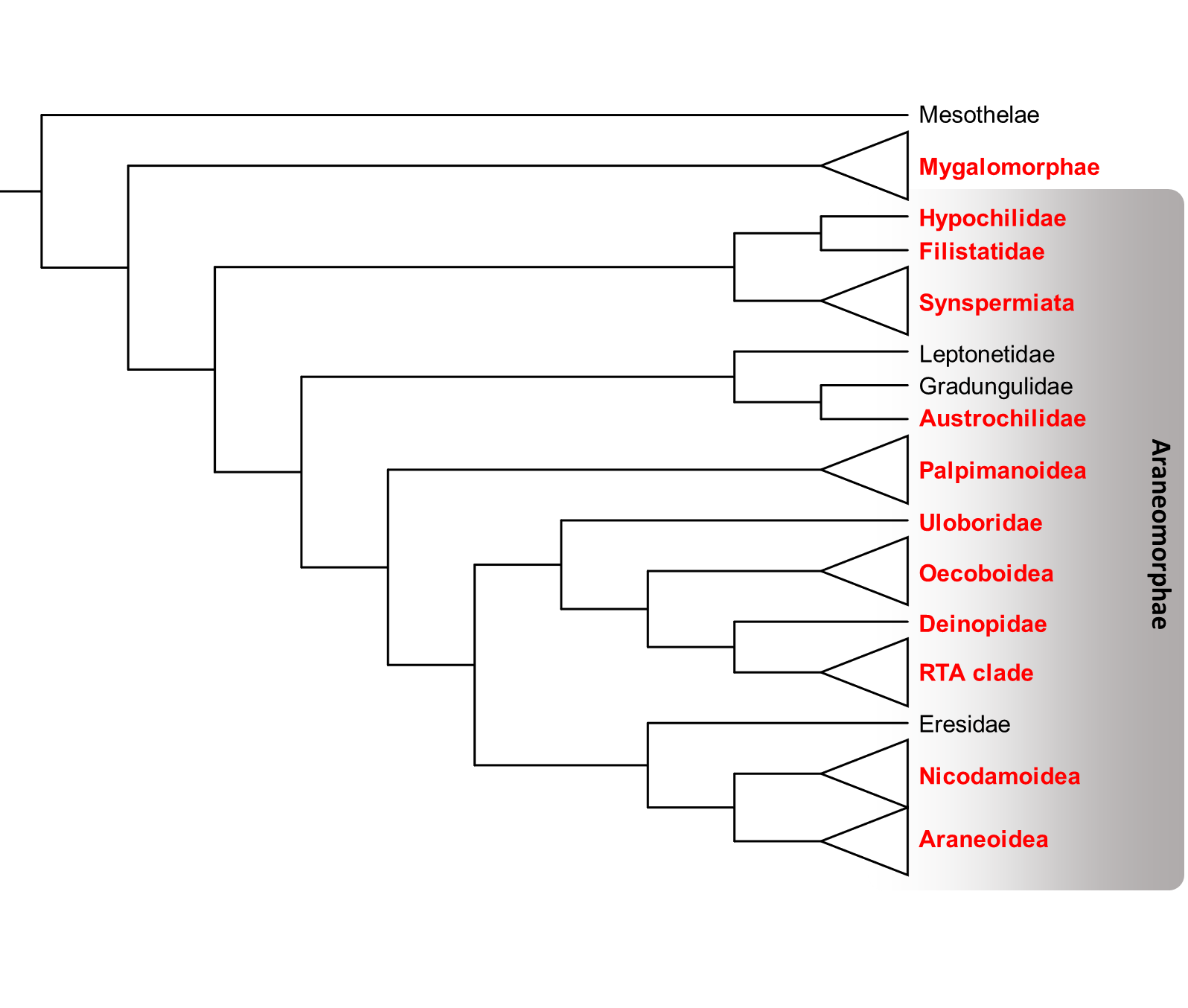


**Figure S17: Phylogenetic tree of the main spiders groups.** Adapted from Kallal et al.(43). In red, groups for which serrated fangs have been clearly observed. For the groups in black, serration has not been observed or the data are not sufficient to evaluate its presence.

**Additional Supplementary Material:**

**Supplementary video 1.** *The cutting of the silk lines*: this high-speed video shows how the spider performed the cutting of its major ampullate silk threads.

**Supplementary video 2.** *Saw movement*: this nocturnal video shows the movements of the spider while sawing Kevlar^®^ fibres.

**Supplementary video 3.** *The cutting of the Kevlar^®^*: this nocturnal video shows the moment when the spider finally cuts the Kevlar^®^ fibre and then collects it with its paws.

**Supplementary video 4.** *3D muscles model*: this video offers an overview of the muscle apparatus of the chelicerae of the spiders.

**Supplementary data sheet.** *Occurrence of serration in spiders*: list of the families, genera and species for which then presence of cheliceral serration is reported.
